# Soluble fermentable dietary fiber attenuates age-related cognitive impairment via neuroimmune and antioxidant modulation: evidence from multilevel analyses in populations and aging mouse models

**DOI:** 10.3389/fimmu.2026.1718673

**Published:** 2026-02-02

**Authors:** Yijie He, Jin Li, Lin Cong, Hui Li, Jiarong Wu, Songlan Liang, Yahui Peng, Yuhong Zhou, Yun Wu

**Affiliations:** 1 Department of Neurology, The Second Affiliated Hospital of Harbin Medical University, Harbin, Heilongjiang, China; 2 Department of Biochemistry and Molecular Biology, School of Basic Medicine, Harbin Medical University, Harbin, Heilongjiang, China; 3 Department of Basic Medicine, Xiamen Medical College, Xiamen, Fujian, China

**Keywords:** aging-related cognitive impairment, gut–brain immune regulation, microglial lineage remodeling, single-nucleus transcriptomics, soluble fermentable dietary fiber

## Abstract

**Background:**

Age-related cognitive impairment (ARCI) is an urgent public health concern with limited therapeutic options. Soluble fermentable dietary fiber (SFDF) is a safe, accessible nutritional factor that may support cognition through microglial remodeling and antioxidant defense, but its dose–response effects and cellular mechanisms remain unclear.

**Methods:**

We combined three levels of evidence (1). In 2,350 older adults from NHANES (2011–2014), weighted regression and spline modeling assessed the association between total dietary fiber intake and cognitive performance. (2) In a D-galactose–induced aging mouse model, inulin supplementation (as a representative SFDF) was tested for effects on behavior, cytokines, and oxidative stress. (3) We analyzed an independent single-nucleus RNA-seq dataset of naturally aged mice receiving a 5% SFDF intervention to characterize microglial state remodeling.

**Results:**

Higher total dietary fiber intake was nonlinearly associated with better cognition, with ~15 g/day as the threshold for maximal benefit. In mice, SFDF improved memory and learning, alleviated anxiety-like behavior, reduced IL-6, TNF-α, and lipid peroxidation, and enhanced antioxidant defenses. Single-nucleus analyses indicated that the 5% SFDF intervention was associated with a shift toward a reparative Mic.7 microglial subtype enriched for immune regulation and oxidative defense programs.

**Conclusions:**

These convergent population, animal, and single-cell findings support a model in which higher total dietary fiber intake is associated with better late-life cognition, and SFDF interventions can attenuate aging-related neuroimmune activation and oxidative stress in experimental systems, highlighting dietary fiber as a scalable nutritional strategy to support healthy cognitive aging.

## Graphical Abstract

**Figure:**
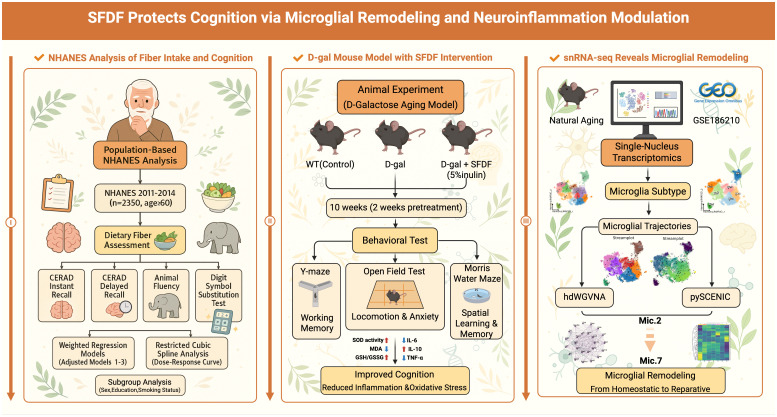
This study adopted a multi-level design to investigate the role of soluble fermentable dietary fiber (SFDF) in age-related cognitive impairment. (I) Population analysis: Using NHANES 2011–2014 data (n=2,350; age ≥60), survey-weighted regression and restricted cubic spline models were applied to examine associations between total dietary fiber intake and multiple cognitive domains, with prespecified stratified analyses by sex, education, and smoking status. (II) A D-galactose–induced accelerated aging mouse model was established to evaluate the effects of SFDF supplementation (5% inulin as a representative SFDF) on behavior, neuroinflammation, and oxidative stress. (III) Mechanistic exploration: Single-nucleus transcriptomic sequencing combined with trajectory inference and network analyses was used to characterize microglial subtypes, lineage transitions, and functional remodeling under SFDF intervention. Created in BioRender. He, Y. (2026) https://BioRender.com/lt3y2t9.

## 1 Introduction

Age-related cognitive impairment (ARCI) is increasingly prevalent with global population aging and represents a major public health challenge (1). ARCI often precedes clinically diagnosed dementia and is associated with reduced quality of life and substantial societal burden (2). Beyond classical neurodegenerative hallmarks, converging evidence implicates chronic low-grade inflammation and oxidative stress as central drivers of cognitive aging. In this context, neuroimmune dysregulation—particularly microglia-mediated immune surveillance and inflammatory remodeling—has emerged as a critical biological axis linking systemic exposures to brain function during aging (3, 4).

Diet is a modifiable determinant of cognitive aging with considerable translational potential (5–7). Dietary fiber has attracted attention not only for its metabolic benefits but also for its capacity to shape the gut–brain–immune axis (8, 9). By altering gut microbial composition and enhancing production of short-chain fatty acids (SCFAs), dietary fiber intake may influence peripheral immune tone, redox balance, blood–brain barrier integrity, and neuroinflammatory signaling (9, 10). Importantly, dietary fiber is not a homogeneous entity but comprises multiple types, most commonly categorized as soluble fermentable dietary fiber (SFDF) and insoluble dietary fiber (ISDF) (11). SFDF is readily utilized by commensal microbes and is more likely to generate bioactive metabolites with immunomodulatory properties, whereas insoluble fiber primarily contributes to intestinal transit and stool bulk (11). These distinctions suggest that SFDF may be particularly relevant to immune-mediated mechanisms underlying cognitive aging (12–14).

Among soluble fermentable dietary fibers, inulin was selected as a representative SFDF for the experimental components of this study. Inulin is a naturally occurring fructan composed primarily of β(2→1)-linked fructose units and is widely present in plant-based foods such as chicory root, Jerusalem artichoke, onions, and garlic (15). As a well-characterized prebiotic, inulin has been extensively used in nutritional and immunological research to model fermentable fiber exposure (16). Its high solubility and fermentability enable consistent modulation of gut microbial composition and SCFA production (11). Importantly, inulin has been shown to influence immune-related pathways, including regulation of inflammatory cytokine signaling and oxidative stress responses (17), which are highly relevant to neuroimmune regulation during aging (18, 19). In addition, the defined chemical structure and high reproducibility of inulin make it particularly suitable for controlled animal experiments, allowing clearer attribution of SFDF-related biological effects compared with heterogeneous dietary fiber mixtures (20). Accordingly, inulin serves as a practical model SFDF for controlled mechanistic testing rather than a proxy for all dietary fiber sources.

Despite strong biological plausibility, several important gaps remain. First, population studies examining dietary fiber intake and cognition are predominantly cross-sectional and often emphasize global associations without systematically characterizing nonlinear dose–response relationships, potential intake plateaus or turning points, or domain-specific cognitive patterns (21–23). Second, cognitive function is multidimensional, yet effect heterogeneity across clinically relevant subgroups—such as sex, educational attainment, and smoking status—has not been comprehensively evaluated in nationally representative aging cohorts. Third, and most relevant to immunology, the central immune cell programs linking dietary fiber exposure to cognitive phenotypes remain insufficiently defined. Microglia, the resident immune cells of the central nervous system, undergo age-dependent transitions from homeostatic surveillance states toward inflammatory and metabolically stressed phenotypes, contributing to synaptic dysfunction and cognitive decline (18, 19). Although single-nucleus transcriptomics has revealed substantial heterogeneity in microglial states during aging and neurodegeneration, nutrient-responsive microglial remodeling and its immunometabolic features remain underexplored.

To address these gaps and enable robust population-level inference, we leveraged data from the National Health and Nutrition Examination Survey (NHANES). NHANES provides standardized 24-hour dietary recall data and harmonized cognitive assessments in older adults, and employs a complex probability-based sampling framework that supports nationally representative estimates when dietary sample weights, strata, and primary sampling units are properly incorporated (24). Notably, the novelty and added value of our NHANES component lie in jointly characterizing domain-specific cognitive outcomes, nonlinear dose–response patterns (including potential plateaus/turning points), and effect heterogeneity through prespecified subgroup analyses (e.g., by sex, educational attainment, and smoking status) within a single, well-characterized, nationally representative aging cohort. This framework provides an interpretable population-level signal and helps identify intake ranges where marginal cognitive gains may plateau, thereby improving translational relevance. In the present study, NHANES is used not as a stand-alone causal dataset, but as a hypothesis-generating and externally generalizable epidemiologic layer that is triangulated with experimental and cellular evidence to strengthen biological plausibility and mechanistic interpretation.

Accordingly, we designed a multilevel, hypothesis-driven study integrating population-based analyses, experimental validation in aging mouse models, and microglia-focused single-nucleus transcriptomics. First, using NHANES 2011–2014, we examined associations between total dietary fiber intake and multiple cognitive domains in older adults, modeled nonlinear dose–response relationships using restricted cubic splines, and evaluated effect heterogeneity across key subgroups. Second, to test biological plausibility and causal effects in an experimental setting, we employed a D-galactose–induced accelerated aging mouse model and evaluated whether inulin, as a representative SFDF, modifies cognitive behavior alongside neuroinflammatory cytokines and oxidative stress markers (25). Third, to elucidate cellular mechanisms, we analyzed an independent single-nucleus RNA sequencing dataset from naturally aged mice receiving dietary fiber intervention, focusing on microglial lineage remodeling and immune-related transcriptional programs. We deliberately combined an accelerated aging intervention model with an independent naturally aged transcriptomic dataset to improve cross-model generalizability of neuroimmune mechanisms. By explicitly distinguishing observational associations from experimental and cellular evidence, this integrative framework aims to provide an immunologically grounded understanding of how soluble fermentable dietary fiber may modulate age-related cognitive impairment.

## 2 Methods

### 2.1 Data sources and study population

This study was based on cross-sectional data from the 2011–2014 cycles of the U.S. National Health and Nutrition Examination Survey (NHANES, https://wwwn.cdc.gov/nchs/nhanes/Default.aspx). NHANES, conducted by the National Center for Health Statistics (NCHS), employs a multistage stratified probability sampling design to collect nationally representative information on the health and nutritional status of the U.S. non-institutionalized civilian population. All participants provided written informed consent, and the survey protocol was approved by the NCHS Institutional Review Board.

Eligibility criteria included age ≥60 years and availability of complete data on two 24-hour dietary recalls, four standardized cognitive assessments, and all key covariates. Exclusions were applied to participants with missing cognitive assessments (n = 716), missing dietary data (n = 200), or incomplete covariates (n = 366). After screening, 2,350 older adults were included in the final analysis.

### 2.2 Assessment of dietary fiber intake

Dietary intake data were obtained through two 24-hour dietary recalls conducted using the U.S. Department of Agriculture’s Automated Multiple-Pass Method (AMPM) by trained interviewers. The first recall was performed in person at the Mobile Examination Center (MEC), and the second was collected via a telephone follow-up. Nutrient data were derived from the Food and Nutrient Database for Dietary Studies (FNDDS). Daily dietary fiber intake was calculated as the two-day mean (g/day) and modeled both as a continuous variable and as quartiles. Nutrient analyses were weighted using Day 1 dietary sample weights (WTDRD1).

### 2.3 Cognitive function assessment

Cognitive performance was measured using four standardized tests from the NHANES cognitive module:

1. CERAD Instant Recall: a learning task involving three trials of 10-word lists, score range 0–30;
2. CERAD Delayed Recall: recall of the word list after a 5-minute delay, score range 0–10;
3. Animal Fluency: enumeration of as many animals as possible within 60 seconds, assessing verbal fluency and executive function;
4. Digit Symbol Substitution Test (DSST): a symbol–number matching task completed in 91 seconds, assessing processing speed and sustained attention, score range 0–133.

All cognitive test scores were analyzed as continuous variables without aggregation or standardization. Participants missing any of the four tests were excluded.

### 2.4 Covariates

Analyses adjusted for a range of sociodemographic, lifestyle, and clinical covariates (26):

1. Age (continuous) and sex (male/female);
2. Race/ethnicity (RIDRETH3 recoded): Mexican American, Other Hispanic, Non-Hispanic White, Non-Hispanic Black, Other/Multiracial (codes 6 and 7 combined);
3. Education level (DMDEDUC2):<9th grade, 9–11th grade, high school/GED, some college/associate degree, and college graduate or above;
4. Socioeconomic status: measured by the Poverty Income Ratio (PIR, continuous);
5. Body mass index (BMI, kg/m²): underweight (<18.5), normal (18.5–24.9), overweight (25.0–29.9), and obese (≥30.0);
6. Smoking status: never, former, or current smoker, derived from SMQ020 and SMQ040;
7. Alcohol consumption: categorized as none, 1–5 times/month, 5–10 times/month, or >10 times/month;
8. Chronic disease history: including diabetes, hypertension, hypercholesterolemia, and cardiovascular disease.

### 2.5 NHANES statistical analysis

All analyses were conducted in R (Version 4.2.1) using the survey(Version 4.4.2) (27), rms(Version 6.8.2) (28), dplyr(Version 1.1.4) (29), and ggplot2(Version 4.0.1) (30) packages. A complex survey design object was constructed using NHANES dietary weights (WTDRD1), stratification variables (SDMVSTRA), and primary sampling units (SDMVPSU) to produce nationally representative estimates among non-institutionalized older U.S. adults. All descriptive and regression analyses incorporated the survey design object (weights/strata/PSU). When pooling the 2011–2012 and 2013–2014 cycles, the Day-1 dietary weights (WTDRD1) were divided by 2 to create 4-year weights, following NCHS analytic guidance for combining two 2-year cycles.

Missing data were handled using a complete-case approach; participants with missing dietary intake, cognitive assessments, or key covariates were excluded from the analytical sample for the corresponding models. To reduce missingness in key dietary variables, fiber intake was calculated as the mean of two 24-h recalls when both days were available; when the Day-2 recall was missing, the Day-1 value was used. All descriptive and regression analyses incorporated the NHANES complex sampling design (sample weights, strata, and PSUs). We acknowledge that complete-case restriction may attenuate representativeness and introduce selection bias if missingness is related to the exposure and/or outcomes; therefore, NHANES findings are interpreted conservatively as survey-weighted associations within the analytic sample, and this is noted as a limitation. Robustness checks focused on model specification and exclusion of extreme fiber intake values, as described below, rather than missing-data correction methods (e.g., inverse-probability weighting or multiple imputation). Prior to modeling, distributions of cognitive outcomes were inspected using histograms and Q–Q plots, and key linear-model assumptions were assessed using residual diagnostics. Cognitive test scores were modeled as continuous outcomes; no transformations were applied because diagnostics did not suggest substantial violations.

Analytical steps included:

1. Descriptive statistics: weighted comparisons of demographic and cognitive characteristics across quartiles of dietary fiber intake;

2. Multivariable linear regression: fiber intake modeled as a continuous variable against each cognitive outcome;

Model 1: adjusted for age;
Model 2: additionally adjusted for sex, race/ethnicity, education, PIR, BMI, smoking, and alcohol use;
Model 3: further adjusted for chronic disease history;

3. Trend analysis: tested linear trends by including the median value of each quartile as a continuous variable;

4. Nonlinear association: examined using restricted cubic spline (RCS) models with knots placed at the 5th, 35th, 65th, and 95th percentiles of the intake distribution (approximately 6.5, 15.4, 23.8, and 36.2 g/day). Statistical evidence for nonlinearity was assessed by testing the nonlinear spline components (two-sided P< 0.05). A “turning point/plateau onset” was interpreted as the intake range where the spline-estimated slope (marginal effect) began to attenuate; this value served as an intepretive reference rather than an arbitrary categorical cut-off.

5. Subgroup and interaction analyses: stratified models by sex, education, and smoking status, with interaction terms to evaluate effect modification;

6. Sensitivity analyses: We assessed robustness by excluding extreme fiber intake values (<5 g/day or >40 g/day) and by re-estimating models using alternative fiber exposure definitions (dietary, supplement-derived, and total fiber), as described above;

7. Multiple outcomes and hypothesis testing: Four cognitive outcomes were prespecified *a priori* and analyzed as distinct cognitive domains. We did not apply a formal family-wise error correction (e.g., Bonferroni) across these four outcomes because they are not repeated measurements of a single construct. Accordingly, interpretation emphasized effect sizes with 95% confidence intervals, consistency in effect direction, and dose–response patterns, together with biological plausibility supported by experimental and single-nucleus transcriptomic evidence, rather than isolated P values.

For subgroup and interaction analyses (including linear interaction terms and RCS-based interaction/nonlinearity tests), P values were adjusted for multiple comparisons using the Benjamini–Hochberg false discovery rate (FDR) procedure, and adjusted P values are reported where applicable.

### 2.6 SFDF intervention in a D-galactose–induced accelerated aging mouse model

A D-galactose–induced accelerated aging paradigm was selected because chronic D-gal administration reproduces key hallmarks of brain aging (oxidative stress/AGE accumulation, neuroinflammation, and reproducible learning–memory deficits) within a practical timeframe for controlled dietary intervention. This experimental layer complements the naturally aged mouse snRNA-seq reanalysis by enabling mechanistic and behavioral validation under standardized conditions.

All experimental procedures were approved by the Animal Ethics Committee of the Second Affiliated Hospital of Harbin Medical University (Approval No. YJSDW2024-019). Male C57BL/6 mice (6–8 weeks old, 18–22 g) were purchased from Cyagen Biosciences Inc. (China). Mice were housed at 22–24°C with 50% ± 10% relative humidity under a 12 h light/dark cycle, with free access to food and water. A 7-day acclimation period preceded the experiment. Inulin was used as a representative soluble fermentable dietary fiber (SFDF) in the mouse experiments and the aging-mouse single-cell dataset referenced in this study; hereafter, we refer to this intervention as SFDF (inulin-based) unless otherwise specified (e.g., when reporting diet composition).

The group size (n = 10 mice per group) was chosen *a priori* based on (i) sample sizes commonly used in D-galactose–induced aging studies assessing cognition and neuroinflammation/oxidative stress endpoints, (ii) feasibility and ethical considerations to minimize animal use while ensuring adequate precision for behavioral and biochemical outcomes, and (iii) our prior experience with variability in these assays. This sample size is within the range reported in comparable interventions and was judged sufficient to detect biologically meaningful differences across multiple endpoints (31–34).

Mice were randomly assigned into three groups (n = 10 per group):

1. Control group (WT): fed a standard AIN-93M diet (5% cellulose) with daily intraperitoneal injections of sterile saline (volume matched);
2. D-gal group: fed the AIN-93M diet with daily intraperitoneal injections of D-galactose (200 mg/kg/day) for 8 consecutive weeks;
3. D-gal + 5% SFDF group: fed a modified diet containing 5% inulin plus 5% cellulose, with daily intraperitoneal injections of D-gal (same as above).

We selected 5% inulin as a commonly used dietary supplementation level in rodent studies to achieve a physiologically meaningful increase in fermentable soluble fiber intake without altering overall energy density or macronutrient composition. To maintain isocaloric conditions and comparable total fiber content, inulin was introduced primarily by substituting an equal proportion of non-fermentable cellulose in the control diet. This design allows isolating the effect of fermentable soluble fiber from total fiber quantity (4, 35–37).

Dietary intervention commenced 2 weeks prior to D-galactose administration and was maintained throughout the subsequent 8-week D-galactose exposure period (total duration: 10 weeks). D-galactose (Sigma–Aldrich, St. Louis, MO, USA; Cat. No. G0750) was used to induce the accelerated aging model. Inulin (YuanYe Bio-Technology Co., Ltd., Shanghai, China; Cat. No. S11143) was used as the soluble fermentable dietary fiber supplement, and the customized experimental diets were formulated by Jiangsu Xietong Pharmaceutical Bioengineering Co., Ltd. (Nanjing, Jiangsu, China).

At the end of the experimental procedures, mice were anesthetized with 3% isoflurane gas (inhalation at 2–3 L/min for approximately 3–5 minutes) until complete loss of the pedal withdrawal reflex was confirmed, indicating a surgical plane of anesthesia and full unconsciousness. Euthanasia was then performed by exsanguination via abdominal aorta blood collection while the animals remained under deep anesthesia. All procedures complied with the AVMA Guidelines for the Euthanasia of Animals (2020) and were approved by the Animal Ethics Committee of the Second Affiliated Hospital of Harbin Medical University. All efforts were made to minimize animal pain and distress.

### 2.7 Behavioral testing

Behavioral assessments were conducted using the Labmaze (Version 5.2.8) automated video-tracking system (Zhongshi Technology, China) to ensure objectivity and standardization (38, 39).

Mice were randomly allocated to experimental groups after acclimatization using a randomized allocation procedure. Group assignments were coded by a researcher not involved in behavioral testing, and animals were identified using coded cage/ID labels during testing. Investigators conducting behavioral testing were blinded to group allocation throughout Y-maze, open field test, and Morris water maze procedures. Behavioral endpoints were extracted automatically by the Labmaze system using coded identities, and statistical analyses were performed with group codes concealed until completion of primary endpoint extraction.

Blinding was applied to the following behavioral endpoints:

1. Y-maze: total arm entries and spontaneous alternation rate;
2. Open field test: total distance traveled, distance traveled in the central zone, time spent in the central zone, and frequency of central-zone entries;
3. Morris water maze: escape latency during training, latency to first cross the former platform location, number of crossings, and time spent in the target quadrant during the probe trial.

#### 2.7.1 Y-maze test

The Y-maze test was performed to evaluate spatial working memory (40). The apparatus consisted of three arms (40 cm × 10 cm × 15 cm) arranged at 120° angles. Mice were acclimated to the laboratory environment for 30 minutes before testing. Each mouse was placed in the center of the maze and allowed to explore freely for 10 minutes (41). Spontaneous alternation was defined as consecutive entries into three different arms. Data from mice with<8 total arm entries were excluded.

The spontaneous alternation percentage was calculated as:


$$Alternation\;rate\;\left(\%\right)=\frac{Number\;of\;alternations}{Total\;entries\;-\;2}\times 100$$

#### 2.7.2 Open field test

The OFT was used to assess locomotor activity and anxiety-like behavior (42). The apparatus was a 500 mm × 500 mm × 350 mm black plastic box with the floor divided into a central zone (250 mm × 250 mm) and a peripheral zone. After 30 minutes of adaptation in the testing room, each mouse was placed in the center and allowed to explore freely for 15 minutes. Increased central-zone activity was interpreted as reduced anxiety.

The Labmaze system automatically recorded the following parameters:

1. total distance traveled;
2. distance traveled in the central zone;
3. time spent in the central zone;
4. frequency of central-zone entries.

#### 2.7.3 Morris water maze

The MWM was conducted to evaluate spatial learning and memory (43). The apparatus was a circular black pool (120 cm diameter, 50 cm depth) filled with opaque water (30 cm depth, 22 ± 1°C) made cloudy with talcum powder. A transparent circular platform (10 cm diameter) was submerged 1 cm below the water surface.

The experiment consisted of three phases: adaptation (Day 1), training (Days 2–6), and probe testing (Day 7).

Training phase: Each mouse underwent four trials per day, released from different quadrants. If the platform was located within 60 s, escape latency was recorded.

Probe trial: On Day 7, the platform was removed. Mice were released from the opposite quadrant, and the following were recorded within 60 s:

1. latency to first cross the former platform location;
2. number of crossings over the platform location;
3. time spent in the target quadrant.

### 2.8 Measurement of inflammatory cytokines (ELISA)

At the end of the experiment, mice were deeply anesthetized, and blood samples were collected from the abdominal aorta. After 30 minutes at room temperature, samples were centrifuged at 3,000 rpm for 10 minutes at 4°C to obtain serum. Concentrations of inflammatory cytokines were measured by ELISA kits according to manufacturer instructions:

1. TNF-α ELISA kit (Jianglai Biotechnology, Shanghai, China; Cat. No. JL10484);
2. IL-6 ELISA kit (Jianglai Biotechnology, Shanghai, China; Cat. No. JL20268);
3. IL-10 ELISA kit (Jianglai Biotechnology, Shanghai, China; Cat. No. JL20242).

Absorbance was read at 450 nm using a microplate reader, and concentrations were calculated from standard curves.

### 2.9 Assessment of oxidative stress markers

Brains were rapidly harvested postmortem and stored in liquid nitrogen. Tissue homogenates were centrifuged at 4°C, and supernatants were analyzed using commercial assay kits:

1. Malondialdehyde (MDA) assay kit (Beyotime Biotechnology, Shanghai, China; Cat. No. S0131M), measured at 532 nm;
2. Superoxide dismutase (SOD) activity assay kit (Beyotime Biotechnology, Shanghai, China; Cat. No. S0101M), measured at 450 nm;
3. Glutathione (GSH) content assay kit (Beyotime Biotechnology, Shanghai, China; Cat. No. S0053), measured at 412 nm.

### 2.10 Preprocessing of snRNA-seq data from naturally aged mouse models

Raw single-cell/nucleus RNA-seq data were obtained from the Gene Expression Omnibus (GEO) under accession number GSE163055 (GEO, https://www.ncbi.nlm.nih.gov/geo/). In the present study, we restricted analyses to the naturally aged mice to align with our aging-focused hypothesis. Specifically, we included two aged whole-brain samples: Aged–Low fiber and Aged–High fiber According to the GEO metadata, mice were switched to a modified AIN-93M diet with 1% cellulose plus either 0% inulin (Low fiber) or 5% inulin (High fiber); therefore, the high-fiber condition represents a 5% inulin intervention in naturally aged mice.

Feature–barcode matrices were generated using the Cell Ranger pipeline and subsequently processed in Python with the *omicverse* package(Version 1.7.9) (44) for quality control. The following criteria were applied to filter single cells:

1. a minimum of 500 unique molecular identifiers (UMIs);
2. detection of at least 250 genes;
3. mitochondrial gene proportion ≤20%;
4. exclusion of cells with abnormally high expression values (extreme outliers).

To minimize doublet contamination, *sccomposite*(Version 1.0.0) (45) was used to identify doublets among highly expressing cells, which were removed at the sample level.

High-quality expression matrices were log-transformed (log1p normalization) and the top 2,000 highly variable genes (HVGs) were selected for downstream analysis. Batch effects were corrected using the *Harmony* algorithm(Version 0.0.1) (46), generating an integrated expression space (X_pca_harmony) for subsequent analyses.

### 2.11 Dimensionality reduction, clustering, and cell type annotation

Following Harmony correction, a neighborhood graph was constructed using *scanpy.pp.neighbors* (n_neighbors = 15, n_pcs = 50). UMAP was applied for dimensionality reduction and visualization. Clustering was performed using the Leiden algorithm, and microglial populations were extracted from the results.

Microglia were identified based on the expression of canonical markers (P2ry12, Cx3cr1, Csf1r, Tmem119) and manually validated against the CellMarker v2.0(CellMarker, http://117.50.127.228/CellMarker/) reference database. Subtype classification was further refined using differential gene expression profiles and transcriptional state stratification.

### 2.12 Pseudotime trajectory inference

To delineate lineage structures and developmental trajectories of microglial subtypes during state transitions, multiple trajectory inference methods were applied, including *Monocle2*(Version 2.34.0) (47), *Monocle3*(Version 1.4.26) (48), *Slingshot*(Version 2.12.0) (49), and *StaVIA*(Version 1.3.3) (50), ensuring cross-validation and robustness.

In Monocle2, log-normalized expression data and HVGs were used to construct a *cellDataSet* object. Dimensionality reduction was performed with DDRTree, followed by minimum spanning tree construction and backbone trajectory fitting. Root states were defined based on clustering or marker gene expression, and pseudotime values were assigned accordingly.

Monocle3 analysis employed Harmony-corrected PCA embeddings and UMAP for graph construction using the *learn_graph* function, followed by pseudotime ordering via *order_cells*. Automatic branch detection was supported, with optional root specification using prior cell-type information.

Slingshot used cluster assignments and UMAP embeddings to build a minimum spanning tree among cluster centroids. Principal curves were then fitted to infer lineages and assign pseudotime values independently across multiple branches, with downstream analysis focused on selected lineages.

StAVIA applied a topological modeling framework based on information flow and state transition probabilities within adjacency graphs. It estimated trajectory entropy and topological confidence, defining lineage directions using shortest state-transition paths, particularly effective for complex branching or nonlinear structures.

For all methods, pseudotime values were normalized to a [0,1] scale to standardize subsequent analyses, including gene dynamics, pathway activity modeling, and identification of key regulators across trajectory branches. The multi-algorithm approach provided comprehensive insights into microglial lineage differentiation, transition nodes, and functional shifts.

### 2.13 High-dimensional weighted gene co-expression network analysis

To investigate co-regulatory gene networks among microglial subtypes, *hdWGCNA*(Version 0.4.8) (51, 52) was applied to single-cell transcriptomic data. This approach identifies cell state–specific co-expression modules and infers putative regulatory genes, thereby elucidating functional regulatory networks.

First, Harmony-corrected expression matrices were used to construct meta-cells via *construct_metacells* based on UMAP spatial proximity, reducing data sparsity and improving correlation stability. Genes expressed in at least 5% of cells were retained using the “fraction” method.

Network construction employed a scale-free topology criterion, selecting the soft-thresholding power yielding R² ≥ 0.85. A signed topological overlap matrix (TOM) was computed, and modules were identified via hierarchical clustering with dynamic tree cutting.

Each module’s eigengene (hME) summarized its expression profile, and module membership (kME) quantified gene centrality. Top 20–30 hub genes from modules of interest were extracted to construct regulatory subnetworks, visualized in *Cytoscape*(Version 3.10.4). Module activity at the single-cell level was scored with AUCell, which calculates enrichment of input gene sets using AUC-based rankings, and mapped onto UMAP embeddings.

Module enrichment across microglial subtypes was assessed using odds ratios, followed by Gene Ontology (GO) annotation with *clusterProfiler*(Version 4.12.6). Functional complementarity among hub genes was evaluated to identify core regulatory frameworks.

### 2.14 Single-cell metabolic modeling via compass

To assess metabolic dynamics during microglial lineage differentiation and state transitions, the *Compass* framework(Version 0.9.10) (53) was applied. Compass integrates gene expression data with genome-scale metabolic networks to estimate cell-level metabolic potential.

Standardized expression matrices were mapped to murine metabolic pathways based on gene–reaction associations, generating a reaction activity score matrix. Compass employed linear programming–based flux optimization to estimate potential pathway activity for each cell.

Analyses focused on pathways related to bile acid metabolism, fatty acid activation, glutathione metabolism, and reactive oxygen species (ROS) clearance. Differences in pathway scores across subtypes and trajectories were assessed to identify stage-specific metabolic shifts.

Cross-analyses integrated Compass-derived high-scoring pathways with hdWGCNA modules to examine overlap with co-expressed gene sets, linking transcriptional regulation to metabolic activity.

### 2.15 Transcription factor regulatory network inference

To reconstruct transcriptional regulatory networks underlying microglial heterogeneity, *pySCENIC*(Version 0.12.1) (54) was applied. This workflow integrates co-expression analysis, motif enrichment, and transcription factor activity scoring to generate cell-level gene regulatory networks (GRNs).

Initially, *GRNBoost2*(Version 1.10.0) (55) identified transcription factors and co-expressed candidate target genes, generating preliminary regulons. RcisTarget was then used to perform motif enrichment analysis within candidate regulons, retaining TF–target interactions supported by conserved binding motifs.

*AUCell*(Version 1.26.0) (56) quantified regulon activity scores per cell, generating a regulon–cell activity matrix for downstream analyses including clustering, heatmaps, subtype comparisons, and visualization.

All analyses were performed in a standardized Linux environment using recommended dependencies and curated motif databases. Regulon activity matrices were subsequently integrated with cell-type annotations, trajectory states, and co-expression modules for downstream regulatory mechanism analyses.

## 3 Results

### 3.1 Baseline characteristics and cognitive performance by dietary fiber intake

Among the 2,350 NHANES participants aged ≥60 years, total dietary fiber intake was significantly associated with multiple baseline characteristics (**Table 1**). Compared with the lowest quartile (Q1), participants in the highest quartile (Q4) exhibited healthier behavioral patterns, higher educational attainment, and more favorable metabolic profiles.

**Table 1:** Characteristics of participants aged 60 years and older from the National Health and Nutrition Examination Survey (NHANES) (2011–2014).

Characteristic	Q1 N = 657 (25%)^1^	Q2 N = 586 (25%)^1^	Q3 N = 562 (25%)^1^	Q4 N = 545 (25%)^1^	P-value^2^
Age					0.3
60-69 years	341 (56%)	294 (52%)	273 (50%)	289 (56%)	
70-79 years	177 (24%)	159 (28%)	158 (28%)	151 (28%)	
80+ years	139 (20%)	133 (20%)	131 (23%)	105 (17%)	
Sex					<0.001
female	383 (63%)	325 (58%)	278 (53%)	207 (39%)	
male	274 (37%)	261 (42%)	284 (47%)	338 (61%)	
Race					<0.001
Non-Hispanic White	319 (77%)	304 (81%)	307 (83%)	270 (80%)	
Non-Hispanic Black	196 (12%)	136 (8.4%)	104 (6.3%)	93 (5.3%)	
Other Hispanic	65 (3.9%)	60 (4.0%)	54 (3.2%)	49 (3.1%)	
Mexican American	37 (2.8%)	44 (3.1%)	49 (3.4%)	71 (4.4%)	
Other/multiracial	40 (4.3%)	42 (3.6%)	48 (4.4%)	62 (7.5%)	
BMI					0.15
Underweight(<18.5)	12 (1.0%)	8 (1.3%)	8 (1.6%)	4 (1.3%)	
Normal(18.5 to <25)	149 (22%)	133 (21%)	148 (27%)	155 (29%)	
Overweight(25 to <30)	231 (37%)	196 (35%)	207 (38%)	197 (36%)	
Obese(30 or greater)	265 (40%)	249 (43%)	199 (33%)	189 (33%)	
Alcohol					0.2
1-5 drinks/month	310 (46%)	270 (47%)	277 (49%)	285 (52%)	
5-10 drinks/month	31 (5.4%)	26 (4.3%)	29 (6.1%)	22 (5.4%)	
10+ drinks/month	87 (16%)	103 (22%)	91 (20%)	94 (20%)	
Non-drinker	219 (33%)	184 (26%)	161 (25%)	141 (22%)	
Smoking					<0.001
Current smoker	136 (20%)	67 (12%)	59 (7.4%)	35 (5.3%)	
Former smoker	230 (35%)	223 (37%)	224 (44%)	230 (42%)	
Never smoker	291 (45%)	296 (52%)	279 (49%)	280 (53%)	
Education					<0.001
Less Than 9th Grade	90 (9.5%)	53 (4.2%)	41 (3.5%)	54 (3.6%)	
9-11th Grade	122 (14%)	78 (11%)	64 (7.3%)	45 (6.7%)	
High School Grad/GED	189 (27%)	144 (25%)	126 (19%)	97 (17%)	
Some College or AA degree	170 (33%)	178 (32%)	174 (34%)	164 (30%)	
College Graduate or above	86 (17%)	133 (28%)	157 (36%)	185 (43%)	
PIR	2.76 (1.55)	3.09 (1.60)	3.32 (1.54)	3.47 (1.52)	<0.001
TC/HDL	3.84 (1.30)	3.72 (1.17)	3.68 (1.18)	3.66 (1.09)	0.3
Cardiovascular disease					0.3
Absence	496 (76%)	452 (77%)	442 (79%)	441 (82%)	
Presence	161 (24%)	134 (23%)	120 (21%)	104 (18%)	
Hypertension					0.032
Absence	207 (35%)	217 (42%)	218 (42%)	237 (49%)	
Presence	450 (65%)	369 (58%)	344 (58%)	308 (51%)	
Diabetes					<0.001
Absence	442 (71%)	426 (76%)	424 (81%)	419 (83%)	
Presence	215 (29%)	160 (24%)	138 (19%)	126 (17%)	

^1^n (unweighted) (%); Mean (SD).

^2^Pearson's X^2: Rao & Scott adjustment; Design-based KruskalWallis test.

Specifically, 43% of individuals in Q4 had a college degree or above, more than 2.5 times the proportion in Q1 (17%; P< 0.001). The Poverty Income Ratio (PIR) also increased with total dietary fiber intake, rising from 2.76 in Q1 to 3.47 in Q4 (P< 0.001), indicating that higher intake was linked to more advantaged socioeconomic status. In terms of lifestyle factors, the prevalence of never smoking increased from 45% in Q1 to 53% in Q4 (P< 0.001), while current smoking declined sharply from 20% to 5.3% (P< 0.001). No significant differences were observed across quartiles for alcohol consumption or BMI categories. Higher total dietary fiber intake was also associated with a reduced burden of chronic conditions, particularly diabetes (29% in Q1 vs. 17% in Q4; P< 0.001) and hypertension (65% vs. 51%; P = 0.032).

Importantly, cognitive performance consistently improved with increasing total dietary fiber intake (**Table 2**). In unadjusted analyses, CERAD Instant Recall scores increased from 18.9 (SD 4.7) in Q1 to 20.4 (SD 4.2) in Q4; Animal Fluency scores rose from 16.9 to 19.3; and DSST scores improved from 49 to 55 (all P< 0.01). Although CERAD Delayed Recall scores showed only borderline significance (6.00 to 6.47; P = 0.065), the trend was consistently upward.

**Table 2:** Distribution of cognitive function scores and impairment prevalence across dietary fiber intake quartiles.

Characteristic	Q1 N = 657(25%)^1^	Q2 N = 586(25%)^1^	Q3 N = 562(25%)^1^	Q4 N = 545(25%)^1^	Value^2^
Instant Recall Score	18.9 (4.7)	20.0 (4.3)	20.0 (4.5)	20.4 (4.2)	0.001
Delayed Recall Score	6.00 (2.33)	6.36 (2.25)	6.39 (2.26)	6.47 (2.19)	0.065
Animal Fluency: Score Total	16.9 (5.1)	18.1 (5.4)	18.8 (5.9)	19.3 (5.6)	<0.001
Digit Symbol: Score Total	49 (17)	53 (17)	54 (16)	55 (15)	0.001
CERAD: Instant Recall					0.006
Absence	440 (72%)	446 (80%)	422 (81%)	410 (83%)	
Presence	217 (28%)	139 (20%)	140 (19%)	133 (17%)	
CERAD: Delayed Recall					0.051
Absence	366 (59%)	362 (67%)	355 (69%)	347 (68%)	
Presence	291 (41%)	224 (33%)	207 (31%)	198 (32%)	
Animal Fluency					<0.001
Absence	408 (73%)	433 (81%)	421 (81%)	424 (84%)	
Presence	249 (27%)	153 (19%)	141 (19%)	121 (16%)	
Digit Symbol					<0.001
Absence	416 (76%)	441 (84%)	435 (87%)	437 (90%)	
Presence	241 (24%)	145 (16%)	127 (13%)	108 (9.8%)	

^1^Mean (SD); n (unweighted) (%).

^2^Design-based Kruskal-Wallis test; Pearson's X^2: Rao & Scott adjustment.

The prevalence of cognitive impairment was inversely related to total dietary fiber intake. In Q1, 28% of participants exhibited impairment in CERAD Instant Recall, compared with 17% in Q4. Similarly, the prevalence of DSST-defined impairment declined from 24% in Q1 to 9.8% in Q4 (both P< 0.01). Animal Fluency impairment decreased from 27% to 16% (P< 0.001).

Overall, higher total dietary fiber intake was a marker of more favorable socioeconomic and health status and was associated with superior cognitive performance and a lower prevalence of impairment across multiple cognitive domains. These findings support further evaluation in multivariable models.

### 3.2 Multivariable regression analyses: robustness of associations after adjustment

**Figure 1:**
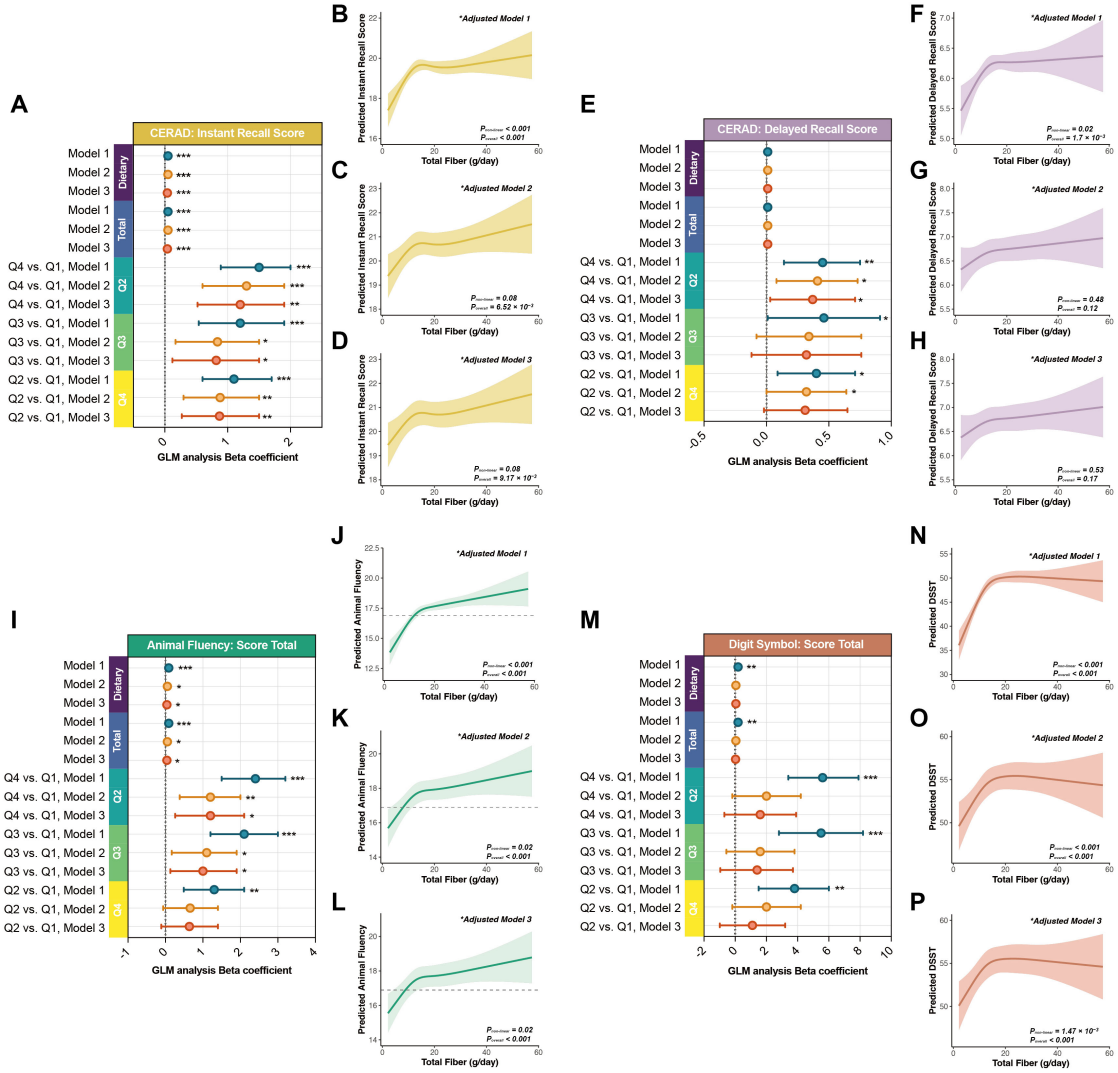
Associations between dietary fiber intake and cognitive performance in adults aged 60 years and older. **(A, E, I, M)** Forest plots depicting linear associations between dietary fiber intake (g/day, as a continuous variable) and four cognitive outcomes—CERAD Instant Recall, CERAD Delayed Recall, Animal Fluency, and DSST—in adults aged ≥60 years. Results are shown under three progressively adjusted models (Model 1–3), with β coefficients and 95% confidence intervals. **(B–D, F–H, J–L, N–P)** Restricted cubic spline (RCS) curves under Models 1 to 3 illustrating potential nonlinear dose–response relationships between dietary fiber intake and cognitive function. *P< 0.05, **P< 0.01, ***P< 0.001.

In multivariable models adjusting for potential confounders, total dietary fiber intake remained positively and nonlinearly associated with cognitive performance across multiple domains (**Figure 1**, **Table 3**). The strongest and most consistent associations were observed for CERAD Instant Recall and Animal Fluency, suggesting that higher intake was associated with better performance in domains involving memory formation, language fluency, and attentional control.

**Table 3:** Linear regression analysis of dietary fiber intake (continuous and quartile categories) with cognitive function scores across multiple adjusted models (beta, 95% CI, p-values).

Comparison (Model)	CERAD: instant recall score	CERAD: delayed recall score	Animal fluency: score total	Digit symbol: score total
Dietary fiber (g/day)				
Model 1	0.05 (0.03, 0.07), p < 0.001	0.01 (0.00, 0.03), p = 0.15	0.09 (0.05, 0.13), p < 0.001	0.18 (0.07, 0.29), p = 0.002
Model 2	0.05 (0.03, 0.06), p < 0.001	0.01 (-0.01, 0.03), p = 0.2	0.05 (0.01, 0.08), p = 0.018	0.05 (-0.04, 0.14), p = 0.3
Model 3	0.04 (0.02, 0.06), p < 0.001	0.01 (-0.01, 0.03), p = 0.3	0.04 (0.01, 0.08), p = 0.028	0.04 (-0.06, 0.13), p = 0.4
Total fiber (g/day)				
Model 1	0.05 (0.03, 0.07), p < 0.001	0.01 (0.00, 0.03), p = 0.2	0.09 (0.05, 0.12), p < 0.001	0.18 (0.07, 0.28), p = 0.002
Model 2	0.05 (0.03, 0.06), p < 0.001	0.01 (-0.01, 0.03), p = 0.2	0.05 (0.01, 0.08), p = 0.014	0.05 (-0.04, 0.14), p = 0.3
Model 3	0.04 (0.02, 0.06), p < 0.001	0.01 (-0.01, 0.03), p = 0.3	0.04 (0.01, 0.08), p = 0.023	0.03 (-0.06, 0.13), p = 0.4
Quartile of total fiber				
Q4 vs Q1, Model 1	1.5 (0.89, 2.0), p < 0.001	0.45 (0.14, 0.75), p = 0.006	2.4 (1.5, 3.2), p < 0.001	5.6 (3.4, 7.9), p < 0.001
Q4 vs Q1, Model 2	1.3 (0.60, 1.9), p < 0.001	0.41 (0.08, 0.73), p = 0.017	1.2 (0.38, 2.0), p = 0.007	2.0 (-0.18, 4.2), p = 0.070
Q4 vs Q1, Model 3	1.2 (0.52, 1.9), p = 0.003	0.37 (0.03, 0.71), p = 0.035	1.2 (0.26, 2.1), p = 0.016	1.6 (-0.70, 3.9), p = 0.2
Q3 vs Q1, Model 1	1.2 (0.54, 1.9), p = 0.001	0.46 (0.01, 0.91), p = 0.045	2.1 (1.2, 3.0), p < 0.001	5.5 (2.8, 8.2), p < 0.001
Q3 vs Q1, Model 2	0.84 (0.17, 1.5), p = 0.018	0.34 (-0.08, 0.76), p = 0.11	1.1 (0.17, 1.9), p = 0.022	1.6 (-0.57, 3.8), p = 0.14
Q3 vs Q1, Model 3	0.82 (0.12, 1.5), p = 0.026	0.32 (-0.12, 0.76), p = 0.14	1.0 (0.13, 1.9), p = 0.028	1.4 (-0.96, 3.7), p = 0.2
Q2 vs Q1, Model 1	1.1 (0.60, 1.7), p < 0.001	0.40 (0.09, 0.71), p = 0.013	1.3 (0.49, 2.1), p = 0.003	3.8 (1.5, 6.0), p = 0.002
Q2 vs Q1, Model 2	0.88 (0.30, 1.5), p = 0.005	0.32 (0.00, 0.64), p = 0.047	0.66 (-0.06, 1.4), p = 0.070	2.0 (-0.18, 4.2), p = 0.070
Q2 vs Q1, Model 3	0.87 (0.27, 1.5), p = 0.009	0.31 (-0.02, 0.65), p = 0.066	0.64 (-0.11, 1.4), p = 0.088	1.1 (-0.99, 3.2), p = 0.3

*Model 1 is adjusted for Age.

*Model 2 is adjusted for Age, Sex, BMI, Alcohol, Smoking, PIR, and Education.

^*^Model 3 is adjusted for Age, Sex, BMI, Alcohol, Smoking, PIR, Education, TC/HDL, Cardiovascular disease, Hypertension, and Diabetes.

In the minimally adjusted model (Model 1, controlling for age only), each additional gram of daily total dietary fiber intake was associated with a 0.05-point increase in CERAD Instant Recall (P< 0.001), a 0.09-point increase in Animal Fluency (P< 0.001), and a 0.18-point increase in DSST (P = 0.002). CERAD Delayed Recall showed a smaller, non-significant change (β = 0.01; P = 0.20).

After further adjustment for sex, BMI, alcohol use, smoking, PIR, and education (Model 2), associations with CERAD Instant Recall (β = 0.05; P< 0.001) and Animal Fluency (β = 0.05; P = 0.014) remained significant and stable, while the DSST effect size attenuated, suggesting that DSST performance may be more sensitive to social and cognitive factors.

In the fully adjusted model (Model 3), which additionally accounted for TC/HDL ratio, cardiovascular disease, hypertension, and diabetes, significant positive associations persisted for CERAD Instant Recall (β = 0.04; P< 0.001) and Animal Fluency (β = 0.04; P = 0.023). Associations with CERAD Delayed Recall and DSST were no longer significant.

When total dietary fiber intake was modeled by quartiles, Q4 consistently outperformed Q1 across all three models, with effect sizes attenuating as covariates were added. For CERAD Instant Recall, the Q4–Q1 difference was +1.5 points in Model 1 (P< 0.001), +1.3 in Model 2 (P< 0.001), and +1.2 in Model 3 (P = 0.003). For Animal Fluency, the advantage was largest in Model 1 (+2.4, P< 0.001) and remained significant in Models 2 and 3 (+1.2; P = 0.007 and 0.016, respectively). CERAD Delayed Recall maintained borderline significance across models (Model 1: +0.45, P = 0.006; Model 2: +0.41, P = 0.017; Model 3: +0.37, P = 0.035). DSST showed significant gains in Model 1 (+5.6, P< 0.001) but was not significant after full adjustment (Model 3: +1.6; P = 0.20).

Restricted cubic spline models further indicated nonlinear dose–response patterns (**Figures 1B–D, F–H, J–L, N–P**). For CERAD Instant Recall and Animal Fluency, cognitive scores increased sharply with total dietary fiber intake up to approximately 15 g/day, after which the curves plateaued, consistent with diminishing marginal gains. This “rapid gain–plateau” pattern was consistent across all three models. In contrast, CERAD Delayed Recall and DSST showed more modest upward trends with greater variability and wider confidence intervals in the mid-to-high intake range.

Collectively, these findings indicate a nonlinear positive association between total dietary fiber intake and cognitive performance, with steeper estimated slopes at lower intake levels and attenuated marginal gains beyond approximately 15 g/day (reported as an interpretive reference point rather than a prespecified cut-off).

### 3.3 Sex-specific subgroup analyses

To further examine whether sex modifies the association between total dietary fiber intake and cognitive function, we performed stratified analyses by sex. The results demonstrated apparent sex-related heterogeneity in effect magnitude and dose–response patterns. Regardless of whether total dietary fiber intake was modeled as a continuous variable or categorized into quartiles, differences in effect size and statistical significance were observed between men and women, suggesting that sex may influence responsiveness along this nutrition–cognition pathway.

**Figure 2:**
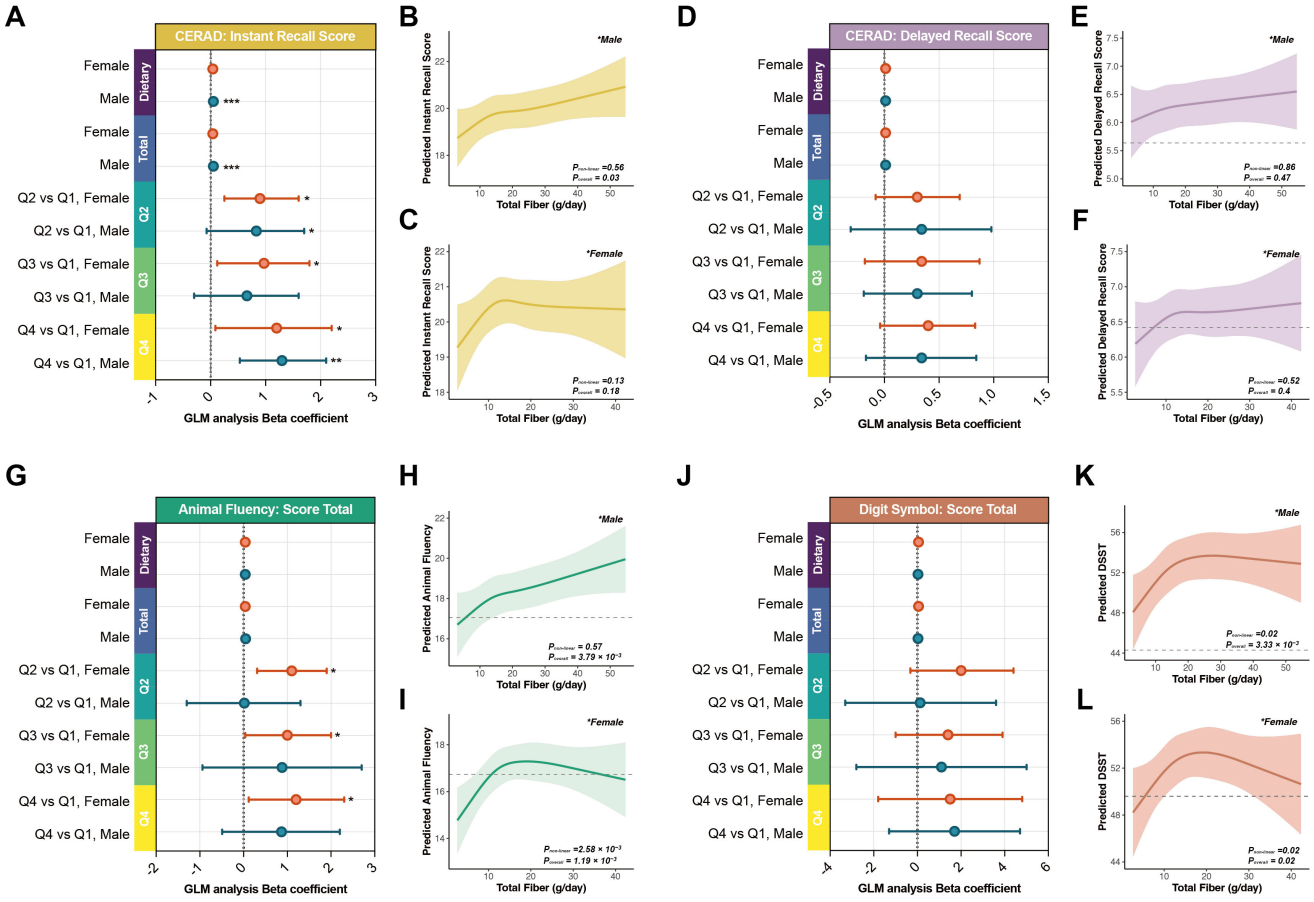
Stratified associations between dietary fiber intake and cognitive function by sex in adults aged 60 years and older. **(A, D, G, J)** Forest plots showing the associations between dietary fiber intake (g/day, continuous) and four cognitive outcomes in male and female participants, based on the fully adjusted Model. β coefficients and 95% confidence intervals are displayed. **(B–C, E–F, H–I, K–L)** Sex-stratified restricted cubic spline (RCS) curves illustrating dose–response relationships between dietary fiber intake and cognition. *P< 0.05, **P< 0.01, ***P< 0.001.

In continuous-variable models, men exhibited more pronounced and statistically significant associations (**Figure 2**, **Table 4**). For each additional gram per day of total dietary fiber intake, CERAD Instant Recall scores in men increased by 0.05 points (P< 0.001), representing the only domain reaching statistical significance. Animal Fluency showed a borderline association (β = 0.05, P = 0.061), whereas CERAD Delayed Recall and DSST demonstrated no meaningful associations (both P > 0.5). In contrast, women displayed smaller effect sizes across domains, with CERAD Instant Recall (β = 0.04, P = 0.077) and Animal Fluency (β = 0.04, P = 0.095) approaching borderline significance, while CERAD Delayed Recall and DSST showed negligible associations.

**Table 4:** Linear regression analysis of dietary fiber intake (continuous and quartile categories) with cognitive function scores stratified by sex (beta, 95% CI, p-values).

Subgroup/Comparison	CERAD: instant recall Score	CERAD: delayed recall score	animal fluency: score total	Digit symbol: score total
Dietary fiber (g/day)				
Male	0.05 (0.03, 0.07), p < 0.001	0.01 (–0.02, 0.03), p = 0.5	0.04 (0.00, 0.09), p = 0.071	0.03 (–0.08, 0.15), p = 0.5
Female	0.04 (0.00, 0.08), p = 0.065	0.01 (–0.01, 0.03), p = 0.2	0.04 (–0.01, 0.08), p = 0.10	0.05 (–0.07, 0.17), p = 0.4
Total fiber (g/day)				
Male	0.05 (0.03, 0.07), p < 0.001	0.01 (–0.02, 0.03), p = 0.6	0.05 (0.00, 0.09), p = 0.061	0.03 (–0.08, 0.14), p = 0.6
Female	0.04 (0.00, 0.08), p = 0.077	0.01 (–0.01, 0.03), p = 0.2	0.04 (–0.01, 0.08), p = 0.095	0.05 (–0.07, 0.17), p = 0.4
Quartile of total fiber				
Q4 vs Q1, Male	1.3 (0.53, 2.1), p = 0.003	0.34 (–0.17, 0.84), p = 0.2	0.87 (–0.49, 2.2), p = 0.2	1.7 (–1.3, 4.7), p = 0.3
Q4 vs Q1, Female	1.2 (0.09, 2.2), p = 0.036	0.40 (–0.04, 0.83), p = 0.072	1.2 (0.12, 2.3), p = 0.033	1.5 (–1.8, 4.8), p = 0.3
Q3 vs Q1, Male	0.66 (-0.30, 1.6), p = 0.2	0.30 (–0.19, 0.80), p = 0.2	0.88 (–0.94, 2.7), p = 0.3	1.1 (–2.8, 5.0), p = 0.5
Q3 vs Q1, Female	0.97 (0.12, 1.8), p = 0.028	0.34 (–0.18, 0.87), p = 0.2	1.0 (0.04, 2.0), p = 0.042	1.4 (–1.0, 3.9), p = 0.2
Q2 vs Q1, Male	0.83 (-0.07, 1.7), p = 0.068	0.34 (–0.31, 0.98), p = 0.3	0.02 (–1.3, 1.3), p > 0.9	0.13 (–3.3, 3.6), p > 0.9
Q2 vs Q1, Female	0.90 (0.25, 1.6), p = 0.011	0.30 (–0.08, 0.69), p = 0.11	1.1 (0.31, 1.9), p = 0.010	2.0 (–0.32, 4.4), p = 0.085

^*^Fully adjusted for Age, BMI, Alcohol, Smoking, PIR, Education, TC/HDL, Cardiovascular disease, Hypertension, and Diabetes.

Quartile-based modeling revealed broader cognitive benefits among women. Compared with Q1, women in Q4 scored significantly higher on CERAD Instant Recall (+1.2, P = 0.003) and Animal Fluency (+1.2, P = 0.033), with CERAD Delayed Recall showing borderline significance (+0.40, P = 0.072). By contrast, in men, significant improvement was observed only for CERAD Instant Recall (+1.3, P = 0.003), while effects for Animal Fluency (+0.87, P = 0.20), CERAD Delayed Recall (+0.34, P = 0.20), and DSST (+1.7, P = 0.30) were directionally consistent but not statistically significant.

Restricted cubic spline (RCS) analyses further illustrated sex-specific dose–response patterns (**Figures 2B–F, H, I, K, L**). For all four cognitive outcomes, inflection points clustered around 15 g/day. Women’s curves exhibited steeper initial slopes with earlier plateauing, suggesting a “rapid response–early saturation” pattern, whereas men’s curves rose more gradually with delayed or absent plateaus.

**Figure 3:**
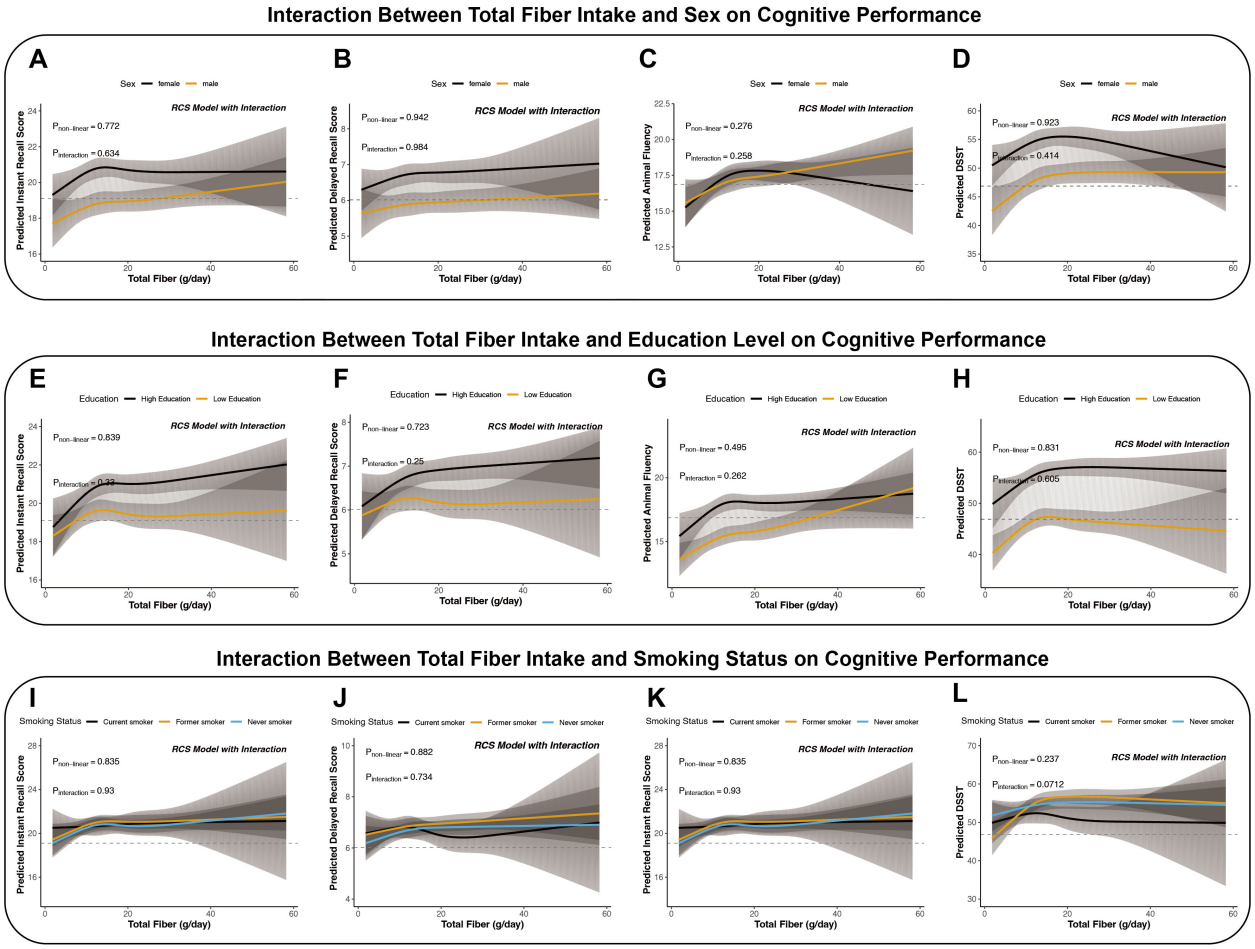
Interaction effects of sex, education, and smoking status on the association between dietary fiber and cognitive function (adults aged 60 years and older). **(A–D)** Show the effect estimates of the dietary fiber × sex interaction terms across four cognitive outcomes. **(E–H)** Show the effect estimates of the dietary fiber × education interaction terms across four cognitive outcomes. **(I–L)** Show the effect estimates of the dietary fiber × smoking status interaction terms across four cognitive outcomes. These P values are unadjusted and are shown for descriptive visualization of effect modification; corresponding multiple-testing–adjusted interaction P values (Benjamini–Hochberg FDR) are reported in **Table 5**.

Formal interaction testing did not provide statistical evidence of sex-based effect modification (**Figures 3A–D**). Neither linear interaction terms nor RCS-based nonlinear interaction terms reached significance after Benjamini–Hochberg false discovery rate (FDR) adjustment (all adjusted P values > 0.40; **Table 5**).

**Table 5:** Interaction effects between total dietary fiber intake and sex, education, and smoking status on cognitive function scores.

Interaction test	CERAD: instant recall score	CERAD: delayed recall score	Animal fluency: score total	Digit symbol: score total
Sex (Male vs. Female)^1^				
Weighted Interaction P-value	0.67441754	0.51485562	0.96156472	0.84801673
RCS Interaction P-value	0.63440533	0.98418358	0.25755571	0.41387406
RCS Nonlinear Interaction P-value	0.77238525	0.94161391	0.27582831	0.92272383
Weighted Interaction adj-P-value	0.96156472	0.96156472	0.96156472	0.96156472
RCS Interaction adj-P-value	0.84587378	0.98418358	0.82774811	0.82774811
RCS Nonlinear Interaction adj-P-value	0.94161391	0.94161391	0.94161391	0.94161391
Education level (High vs. Low)^2^				
Weighted Interaction P-value	0.08587004	0.59640383	0.84120503	0.20396165
RCS Interaction P-value	0.33002173	0.24957387	0.26171702	0.60477335
RCS Nonlinear Interaction P-value	0.83893669	0.72252231	0.49547353	0.83070042
Weighted Interaction adj-P-value	0.34348014	0.79520511	0.84120503	0.4079233
RCS Interaction adj-P-value	0.44002898	0.44002898	0.44002898	0.60477335
RCS Nonlinear Interaction adj-P-value	0.83893669	0.83893669	0.83893669	0.83893669
Smoking status^3^				
Weighted Interaction P-value	0.41926555	0.21573781	0.31238636	0.02576222
RCS Interaction P-value	0.93016873	0.73372866	0.41285055	0.0711553
RCS Nonlinear Interaction P-value	0.83540089	0.8824088	0.27601208	0.23705446
Weighted Interaction adj-P-value	0.41926555	0.41651514	0.41651514	0.10304888
RCS Interaction adj-P-value	0.93016873	0.93016873	0.82570109	0.2846212
RCS Nonlinear Interaction adj-P-value	0.8824088	0.8824088	0.55202416	0.55202416

^1^Models adjusted for Age, Smoke, BMI, Alcohol, Education, PIR, TC/HDL, Cardiovascular disease, Hypertension, and Diabetes.

^2^Models adjusted for Age, Sex, Smoke, BMI, Alcohol, PIR, TC/HDL, Cardiovascular disease, Hypertension, and Diabetes.

^3^Models adjusted for Age, Sex, Education, BMI, Alcohol, PIR, TC/HDL, Cardiovascular disease, Hypertension, and Diabetes.

### 3.4 Education-stratified analyses

**Figure 4:**
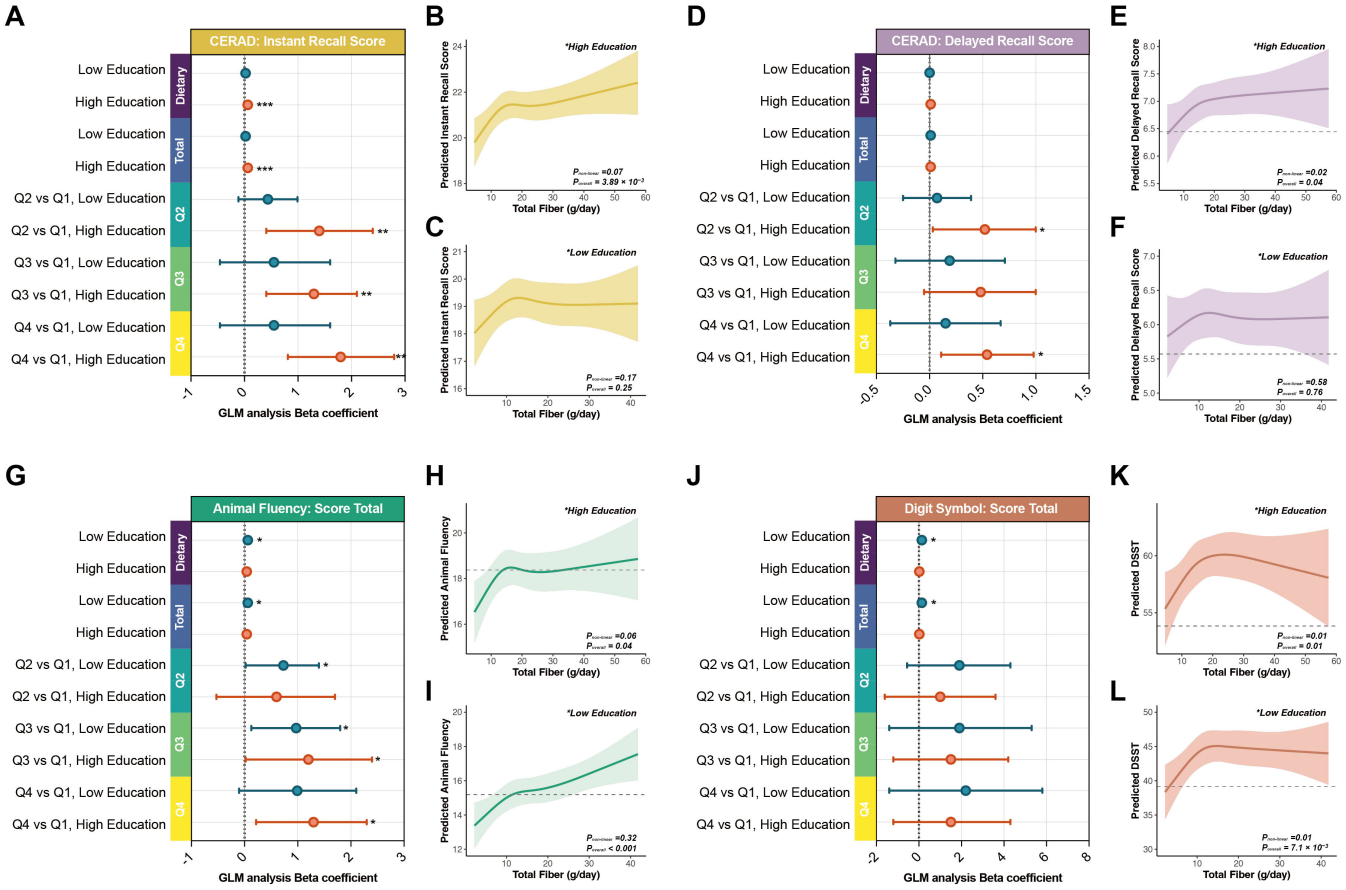
Stratified associations between dietary fiber intake and cognition by education level in adults aged 60 years and older. **(A, D, G, J)** Forest plots showing the associations between dietary fiber intake and cognitive scores in participants with high vs. low education, based on fully adjusted Model. **(B–C, E–F, H–I, K–L)** Education-stratified RCS curves exploring differential dose–response patterns. *P< 0.05, **P< 0.01, ***P< 0.001.

To assess the influence of cognitive reserve on the relationship between total dietary fiber intake and cognitive function, we conducted subgroup analyses stratified by educational attainment, categorized as low education (high school or below) and high education (college or above) (**Figure 4**, **Table 6**).

**Table 6:** Linear regression analysis of dietary fiber intake (continuous and quartile categories) with cognitive function scores stratified by educational level (beta, 95% CI, p-values).

Subgroup/Comparison	CERAD: instant recall score	CERAD: delayed recall score	Animal fluency: score total	Digit symbol: score total
Dietary fiber (g/day)				
High Education	0.06 (0.04, 0.08), p < 0.001	0.01 (–0.01, 0.03), p = 0.3	0.04 (–0.01, 0.09), p = 0.085	0.02 (–0.09, 0.12), p = 0.7
Low Education	0.02 (–0.02, 0.06), p = 0.3	0.00 (–0.02, 0.03), p = 0.7	0.06 (0.00, 0.11), p = 0.035	0.14 (0.01, 0.27), p = 0.042
Total fiber (g/day)				
High Education	0.06 (0.03, 0.08), p < 0.001	0.01 (–0.01, 0.03), p = 0.4	0.04 (0.00, 0.09), p = 0.069	0.02 (–0.09, 0.12), p = 0.8
Low Education	0.02 (–0.02, 0.06), p = 0.3	0.01 (–0.02, 0.03), p = 0.6	0.06 (0.00, 0.11), p = 0.037	0.14 (0.00, 0.27), p = 0.046
Quartile of total fiber				
Q4 vs Q1, High Education	1.8 (0.81, 2.8), p = 0.001	0.54 (0.11, 0.98), p = 0.017	1.3 (0.22, 2.3), p = 0.021	1.5 (–1.2, 4.3), p = 0.2
Q4 vs Q1, Low Education	0.55 (–0.46, 1.6), p = 0.3	0.15 (–0.37, 0.67), p = 0.6	0.99 (–0.10, 2.1), p = 0.072	2.2 (–1.4, 5.8), p = 0.2
Q3 vs Q1, High Education	1.3 (0.41, 2.1), p = 0.006	0.48 (–0.05, 1.0), p = 0.074	1.2 (0.02, 2.4), p = 0.047	1.5 (–1.2, 4.2), p = 0.3
Q3 vs Q1, Low Education	0.55 (–0.46, 1.6), p = 0.3	0.19 (–0.32, 0.71), p = 0.4	0.97 (0.13, 1.8), p = 0.026	1.9 (–1.4, 5.3), p = 0.2
Q2 vs Q1, High Education	1.4 (0.41, 2.4), p = 0.008	0.52 (0.03, 1.0), p = 0.037	0.60 (–0.53, 1.7), p = 0.3	1.0 (–1.6, 3.6), p = 0.4
Q2 vs Q1, Low Education	0.44 (–0.11, 0.99), p = 0.11	0.07 (–0.25, 0.39), p = 0.6	0.73 (0.02, 1.4), p = 0.045	1.9 (–0.55, 4.3), p = 0.12

^*^Fully adjusted for Age, Sex, BMI, Alcohol, Smoking, PIR, TC/HDL, Cardiovascular disease, Hypertension, and Diabetes.

When modeled as a continuous variable, higher education was associated with stronger associations and steeper effect gradients. In the high education group, each 1 g/day increase in total dietary fiber intake corresponded to a significant improvement in CERAD Instant Recall (β = 0.06, P< 0.001) and a borderline improvement in Animal Fluency (β = 0.04, P = 0.069), while CERAD Delayed Recall and DSST did not reach statistical significance. In contrast, the low education group showed weaker associations for CERAD Instant Recall (β = 0.02, P = 0.30) but significant associations with Animal Fluency (β = 0.06, P = 0.037) and DSST (β = 0.14, P = 0.046).

Quartile-based analyses further highlighted education-related differences. In fully adjusted models, participants with higher education in Q4 demonstrated significant gains in CERAD Instant Recall (+1.8, P = 0.001), Animal Fluency (+1.3, P = 0.021), and CERAD Delayed Recall (+0.54, P = 0.017), whereas DSST showed a nonsignificant increase (+1.5, P = 0.20). In the low education group, only Animal Fluency approached significance (+0.99, P = 0.072), with other domains remaining nonsignificant.

RCS analyses revealed that both education groups exhibited inflection points around 15 g/day, but with divergent trajectories (**Figures 4B–F, H, I, K, L**). Participants with higher education showed steeper slopes and sustained gains beyond 15 g/day, whereas those with lower education displayed flatter curves with earlier plateauing.

However, interaction analyses did not support a statistically significant modifying effect of education level (**Figures 3E–H**). After Benjamini–Hochberg FDR adjustment, neither linear nor RCS-based nonlinear interaction terms were significant (adjusted P values > 0.44; **Table 5**).

### 3.5 Subgroup analyses by smoking status

**Figure 5:**
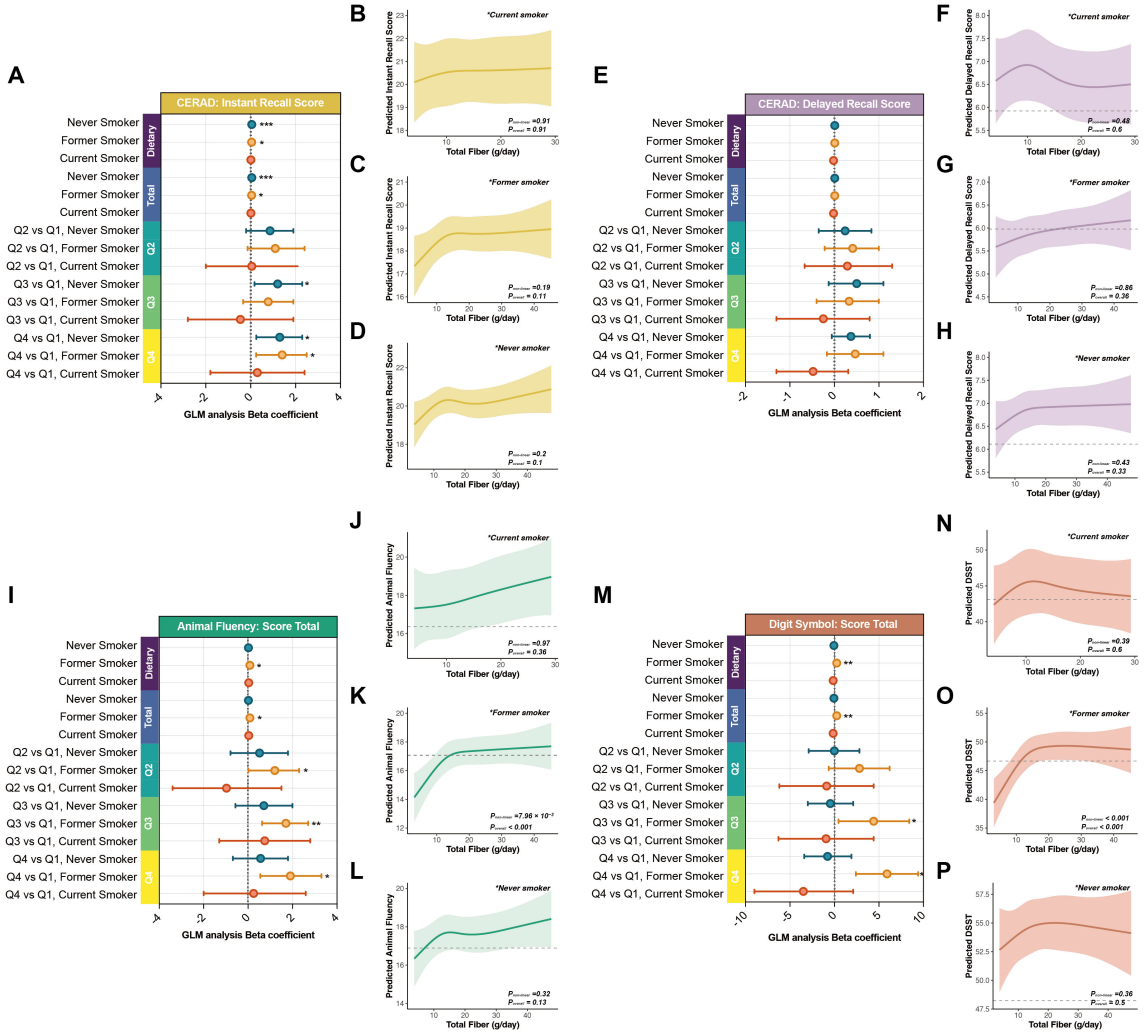
Stratified associations between dietary fiber intake and cognition by smoking status in adults aged 60 years and older. **(A, E, I, M)** Forest plots showing associations between dietary fiber intake and cognitive function in current, former, and never smokers, based on fully adjusted Model. **(B–D, F–H, J–L, N–P)** RCS curves stratified by smoking status demonstrating dose–response heterogeneity. *P< 0.05, **P< 0.01, ***P< 0.001.

Smoking status, a key determinant of systemic inflammation and neurocognitive health, may influence the association between total dietary fiber intake and cognitive performance. We therefore performed stratified analyses by smoking status (never smokers, former smokers, and current smokers) (**Figure 5**, **Table 7**).

**Table 7:** Linear regression analysis of dietary fiber intake (continuous and quartile categories) and cognitive function scores stratified by smoking status (beta, 95% CI, p-values).

Subgroup/Comparison	CERAD: instant recall score	CERAD: delayed recall score	Animal fluency: score total	Digit symbol: score total
Dietary fiber (g/day)				
Current Smoker	0.01 (-0.07, 0.09), p = 0.7	-0.02 (-0.05, 0.01), p = 0.2	0.03 (-0.04, 0.10), p = 0.3	-0.15 (-0.36, 0.06), p = 0.2
Former Smoker	0.04 (0.00, 0.08), p = 0.036	0.01 (-0.01, 0.04), p = 0.3	0.08 (0.02, 0.14), p = 0.012	0.26 (0.10, 0.41), p = 0.003
Never Smoker	0.05 (0.02, 0.07), p < 0.001	0.01 (-0.01, 0.03), p = 0.4	0.02 (-0.02, 0.05), p = 0.3	-0.06 (-0.15, 0.04), p = 0.2
Total fiber (g/day)				
Current Smoker	0.01 (-0.07, 0.09), p = 0.7	-0.02 (-0.05, 0.01), p = 0.2	0.03 (-0.04, 0.10), p = 0.3	-0.15 (-0.36, 0.06), p = 0.2
Former Smoker	0.04 (0.00, 0.08), p = 0.047	0.01 (-0.01, 0.04), p = 0.4	0.08 (0.02, 0.14), p = 0.014	0.25 (0.10, 0.40), p = 0.003
Never Smoker	0.05 (0.02, 0.07), p < 0.001	0.01 (-0.01, 0.03), p = 0.4	0.02 (-0.02, 0.05), p = 0.3	-0.06 (-0.15, 0.04), p = 0.2
Quartile of total fiber				
Q4 vs Q1, Current Smoker	0.30 (-1.8, 2.4), p = 0.8	-0.48 (-1.3, 0.31), p = 0.2	0.25 (-2.0, 2.6), p = 0.8	-3.5 (-9.0, 2.1), p = 0.2
Q4 vs Q1, Former Smoker	1.4 (0.24, 2.5), p = 0.021	0.47 (-0.17, 1.1), p = 0.14	1.9 (0.55, 3.3), p = 0.010	5.9 (2.4, 9.4), p = 0.003
Q4 vs Q1, Never Smoker	1.3 (0.25, 2.3), p = 0.018	0.37 (-0.06, 0.80), p = 0.083	0.56 (-0.68, 1.8), p = 0.3	-0.77 (-3.4, 1.9), p = 0.5
Q3 vs Q1, Current Smoker	-0.46 (-2.8, 1.9), p = 0.7	-0.25 (-1.3, 0.79), p = 0.6	0.75 (-1.3, 2.8), p = 0.5	-0.94 (-6.3, 4.4), p = 0.7
Q3 vs Q1, Former Smoker	0.78 (-0.34, 1.9), p = 0.2	0.33 (-0.40, 1.0), p = 0.4	1.7 (0.63, 2.7), p = 0.004	4.4 (0.45, 8.4), p = 0.032
Q3 vs Q1, Never Smoker	1.2 (0.17, 2.3), p = 0.026	0.50 (-0.13, 1.1), p = 0.11	0.71 (-0.57, 2.0), p = 0.3	-0.46 (-3.0, 2.1), p = 0.7
Q2 vs Q1, Current Smoker	0.05 (-2.0, 2.1), p > 0.9	0.29 (-0.67, 1.3), p = 0.5	-0.97 (-3.4, 1.5), p = 0.4	-0.87 (-6.2, 4.4), p = 0.7
Q2 vs Q1, Former Smoker	1.1 (-0.14, 2.4), p = 0.077	0.41 (-0.22, 1.0), p = 0.2	1.2 (0.02, 2.3), p = 0.047	2.8 (-0.65, 6.2), p = 0.10
Q2 vs Q1, Never Smoker	0.86 (-0.20, 1.9), p = 0.10	0.24 (-0.35, 0.83), p = 0.4	0.52 (-0.79, 1.8), p = 0.4	-0.01 (-2.9, 2.8), p > 0.9

*Fully adjusted for Age, Sex, BMI, Alcohol, Education, PIR, TC/HDL, Cardiovascular disease, Hypertension, and Diabetes.

In continuous-variable models, former smokers exhibited the most pronounced associations. Each additional gram per day of total dietary fiber intake was associated with a 0.04-point increase in CERAD Instant Recall (P = 0.047) and a 0.08-point increase in Animal Fluency (P = 0.014). CERAD Delayed Recall showed a nonsignificant positive association (β = 0.01, P = 0.40). Notably, DSST demonstrated a significant inverse association (β = −0.25, P = 0.003). Never smokers showed a significant association only for CERAD Instant Recall (β = 0.05, P< 0.001), whereas current smokers exhibited no significant associations across cognitive domains.

Quartile-based analyses further underscored smoking-related heterogeneity. Former smokers in Q4 scored significantly higher than those in Q1 on CERAD Instant Recall (+1.4, P = 0.021), Animal Fluency (+1.9, P = 0.010), and DSST (+5.9, P = 0.003). Among never smokers, Q4 participants showed a significant gain in CERAD Instant Recall (+1.3, P = 0.018) and a borderline improvement in CERAD Delayed Recall (+0.37, P = 0.083). No significant Q4–Q1 differences were observed among current smokers.

RCS analyses revealed distinct nonlinear dose–response structures across smoking groups (**Figures 5B–D, F–H, J–L, N–P**). Former smokers showed steep slopes up to 15–20 g/day followed by plateauing, whereas never smokers exhibited moderate slopes with limited marginal gains. Current smokers displayed flat curves with wide confidence intervals.

Interaction testing identified a nominal interaction signal for DSST by smoking status (P = 0.026), which did not remain significant after Benjamini–Hochberg FDR adjustment (adjusted P = 0.10). RCS-based nonlinear interaction terms were also nonsignificant after adjustment (adjusted P = 0.55; **Table 5**), indicating that visual subgroup differences were not supported by formal interaction testing (**Figures 3I–L**).

### 3.6 SFDF intervention significantly improves cognitive and behavioral deficits in D-galactose–induced aging mice

**Figure 6:**
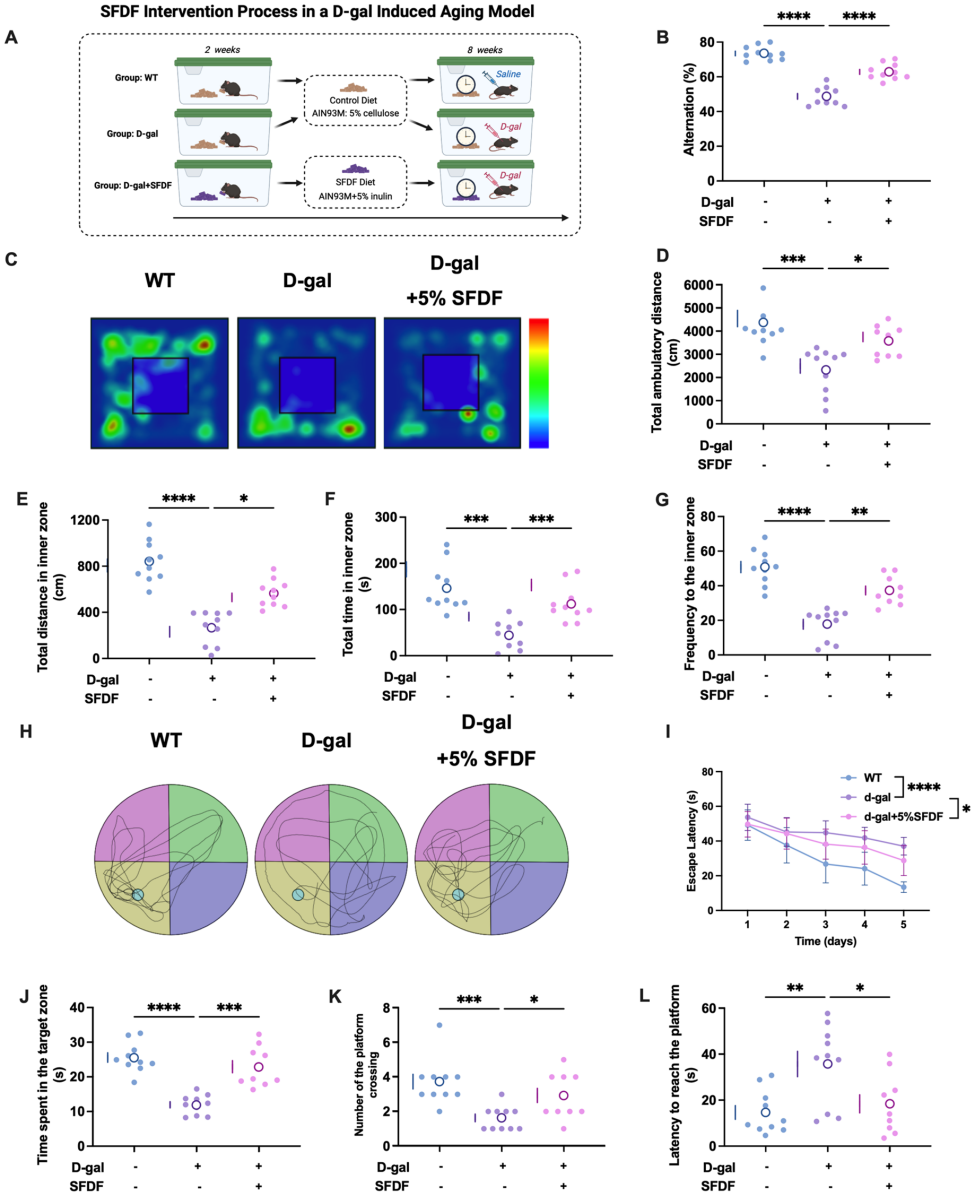
SFDF intervention alleviates cognitive and behavioral deficits in D-galactose-induced aging mice. **(A)** Schematic of the experimental timeline: mice received an SFDF diet (5% inulin, a representative SFDF) starting 2 weeks prior to D-gal injection and continuing throughout the 8-week induction period. Created in BioRender. He, Y. (2026) https://BioRender.com/w5t51f4. **(B)** Y-maze test showed that D-gal significantly reduced spontaneous alternation, which was rescued by SFDF treatment. **(C)** Open field test trajectory maps showed decreased central activity in the D-gal group, indicative of heightened anxiety. **(D–G)** Quantitative metrics revealed reduced total distance, center distance, center time, and center entries in the D-gal group; these deficits were significantly improved by SFDF. **(H)** Morris water maze trajectories revealed more focused searching in the target quadrant in the SFDF group. **(I–L)** Water maze performance indicated prolonged escape latency, shorter target quadrant time, fewer platform crossings, and delayed first arrival in the D-gal group; SFDF treatment significantly improved these learning and memory measures.*P< 0.05, **P< 0.01, ***P< 0.001, ****P< 0.0001.

Building on the population-level findings, we further evaluated the potential role of SFDF in age-related neurofunctional impairment in an experimental setting. To this end, we established a D-galactose (D-gal)-induced accelerated aging mouse model, which recapitulates systemic oxidative stress and aging-like neurobehavioral deficits. In this design, inulin was used as a representative SFDF, and a diet supplemented with 5% SFDF was introduced two weeks prior to D-gal administration and continued throughout the 8-week induction period, forming an integrated “prevention plus treatment” intervention strategy (**Figure 6A**). At the end of the intervention, a battery of behavioral tests was performed to systematically evaluate changes in motor function, anxiety-like behaviors, and cognitive performance, thereby providing experimental support for the population-based observations.

In the Y-maze test, D-gal administration markedly reduced the spontaneous alternation rate (**Figure 6B**, P< 0.0001), indicating impaired working memory. SFDF supplementation significantly improved this parameter (P< 0.0001), demonstrating a clear protective effect on short-term memory.

In the open field test, D-gal treatment resulted in sparse exploratory trajectories in the central zone (**Figure 6C**), reflecting reduced exploratory drive and heightened anxiety-like behavior. Quantitative analyses confirmed significant declines across multiple indices, including total distance traveled (**Figure 6D**), central-zone distance (**Figure 6E**), central-zone duration (**Figure 6F**), and frequency of central-zone entries (**Figure 6G**) (all P values<0.01 to<0.0001). SFDF intervention significantly improved all these parameters (P< 0.01), indicating mitigation of both motor retardation and affective disturbance.

In the Morris water maze, D-gal-treated mice exhibited significantly prolonged escape latency during training (**Figure 6I**, P< 0.0001), indicating impaired spatial learning. SFDF treatment significantly shortened escape latency (P< 0.05), suggesting enhanced learning capacity. In the probe trial, D-gal mice spent less time in the target quadrant (**Figure 6J**), crossed the former platform location fewer times (**Figure 6K**), and required longer to locate the platform initially (**Figure 6L**), indicating deficits in memory retention (P< 0.01 to P< 0.001). SFDF supplementation significantly improved all of these outcomes. Trajectory analyses further confirmed that SFDF-treated mice exhibited more focused search paths within the target quadrant (**Figure 6H**), corroborating its beneficial effect on memory retention.

Collectively, SFDF intervention significantly attenuated D-gal-induced deficits in working memory, spatial learning, locomotor activity, and anxiety-like behaviors, supporting its multidimensional neuroprotective effects in this accelerated aging model.

### 3.7 SFDF attenuates D-gal-induced oxidative stress and neuroinflammation

To further elucidate mechanisms associated with SFDF’s behavioral effects, we evaluated biomarkers of neuroinflammation and oxidative stress.

**Figure 7:**
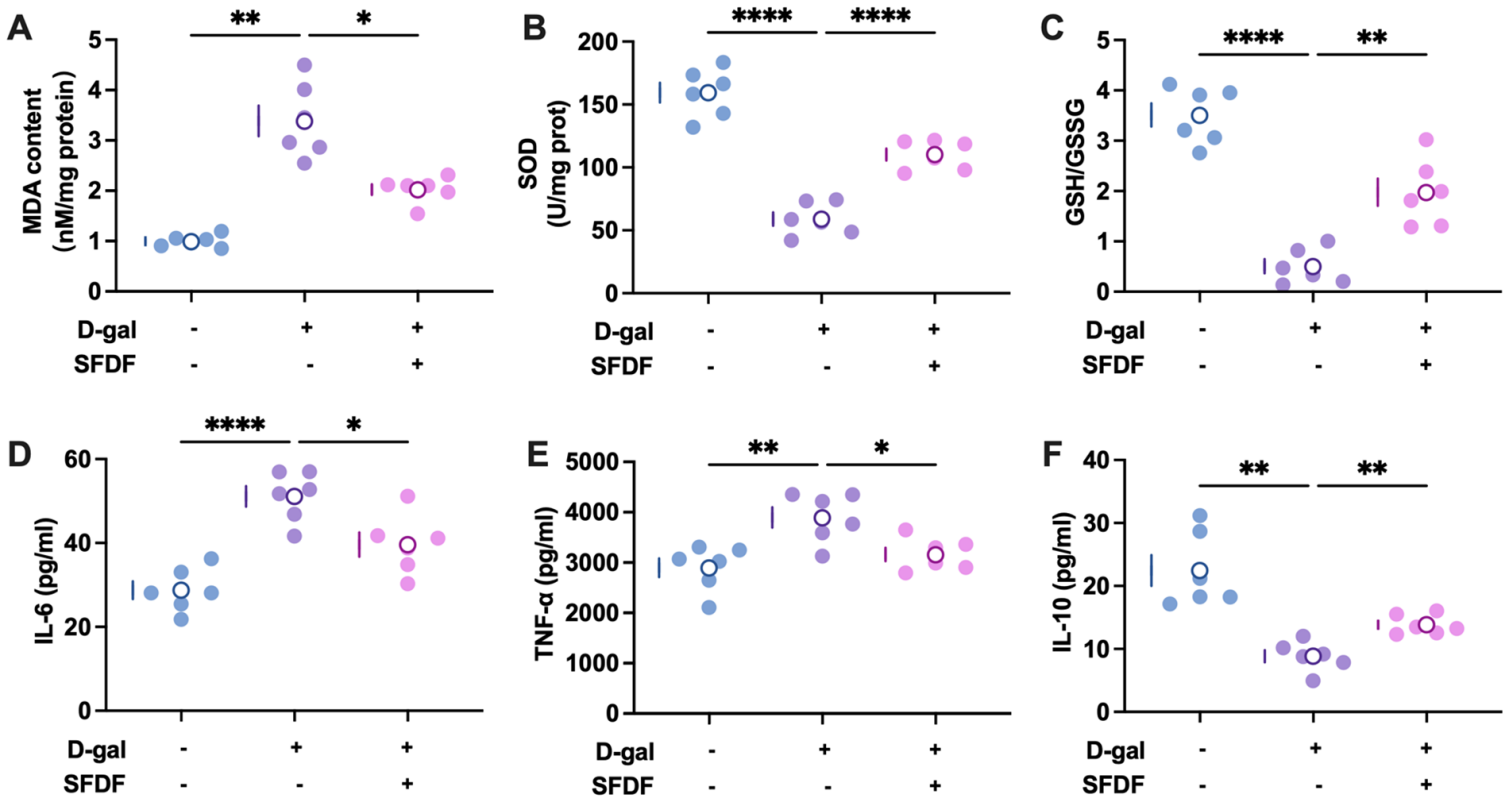
SFDF attenuates D-gal-induced neuroinflammation and oxidative stress. **(A–C)** D-gal administration led to reduced GSH/GSSG ratio, increased MDA levels, and diminished SOD activity, indicating oxidative stress; these abnormalities were significantly reversed by SFDF treatment. **(D–F)** SFDF significantly suppressed D-gal-induced increases in brain pro-inflammatory cytokines (IL-6, TNF-α) and restored the level of the anti-inflammatory cytokine IL-10.*P< 0.05, **P< 0.01, ****P< 0.0001.

D-gal administration significantly upregulated pro-inflammatory cytokines in brain tissue, particularly IL-6 and TNF-α (**Figures 7D, E**), with elevations reaching high significance (P< 0.001 to P< 0.0001). These results indicate robust neuroinflammatory activation induced by D-gal. SFDF supplementation markedly suppressed the expression of these cytokines (P< 0.01 to P< 0.001) and increased IL-10 levels (**Figure 7F**), consistent with an anti-inflammatory shift that may contribute to improved behavioral performance.

Oxidative stress analyses further confirmed profound redox imbalance following D-gal treatment. Specifically, the glutathione reduced/oxidized ratio (GSH/GSSG) was significantly decreased (**Figure 7C**; P< 0.0001), malondialdehyde (MDA) was markedly elevated (**Figure 7A**; P< 0.001), and superoxide dismutase (SOD) activity was significantly reduced (**Figure 7B**; P< 0.0001). SFDF treatment effectively restored the GSH/GSSG ratio, reduced MDA levels, and significantly enhanced SOD activity (P< 0.01 to P< 0.0001), indicating improved antioxidant capacity.

Taken together, SFDF not only ameliorated D-gal-induced behavioral and cognitive deficits but also concurrently suppressed neuroinflammatory activation and oxidative damage, supporting coordinated anti-inflammatory and antioxidative actions.

### 3.8 SFDF intervention reshapes microglial subtype composition and functional states in a natural aging model

To further elucidate immune-cell mechanisms potentially responsive to SFDF in aging, we analyzed single-cell RNA sequencing data from the brains of naturally aged mice receiving dietary fiber intervention. We compared microglial subtype composition, functional states, and cell-cycle dynamics between a low-fiber group and a high-fiber group.

**Figure 8:**
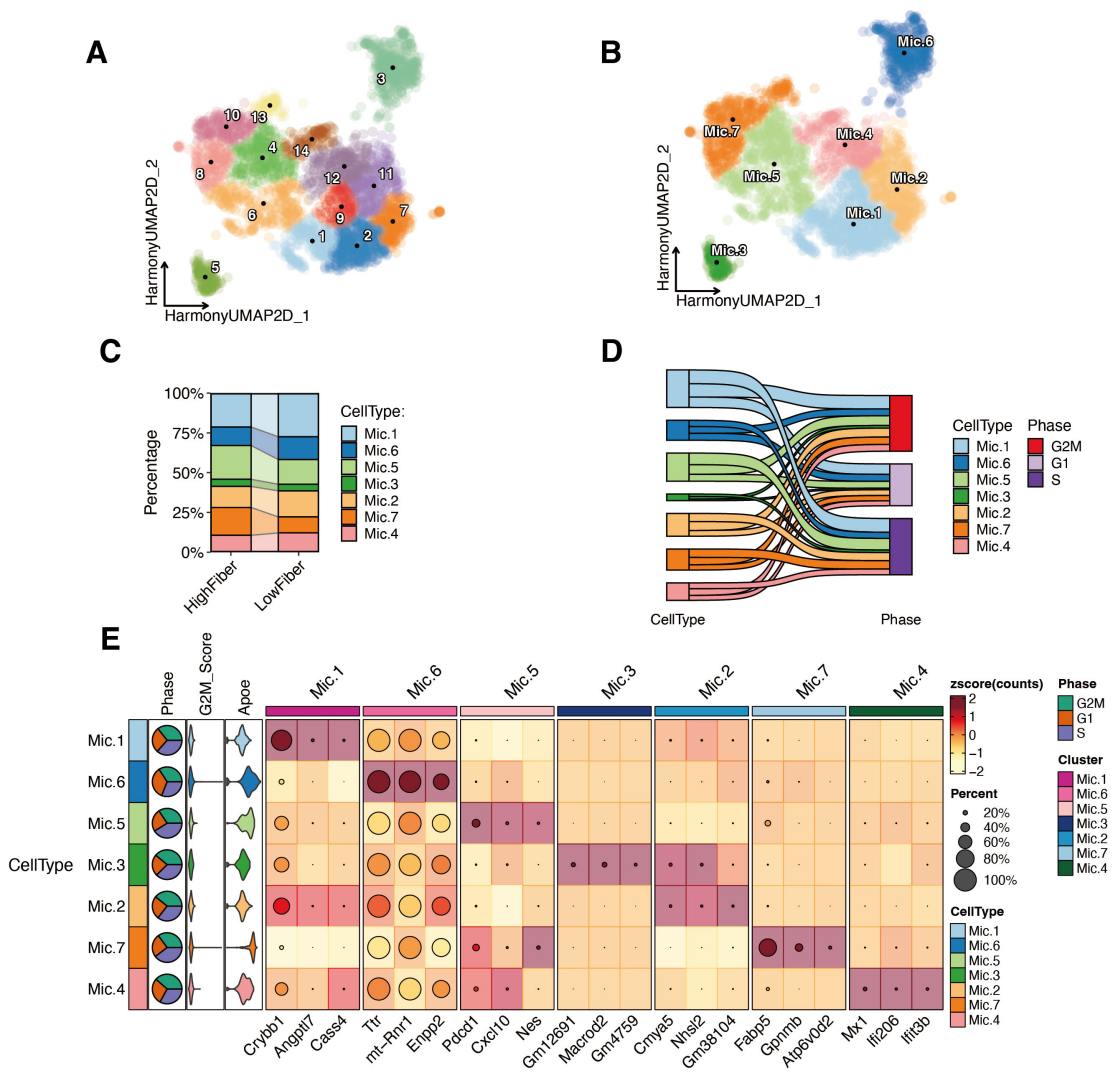
SFDF intervention reshapes microglial lineage composition and functional states in naturally aged mice. **(A)** Fourteen microglial clusters were identified using the Leiden clustering algorithm. **(B)** Based on marker gene expression patterns and literature annotation, these clusters were classified into seven functionally distinct microglial subtypes (Mic.1–Mic.7). **(C)** Comparative analysis of group-wise proportions showed significant enrichment of Mic.5 and Mic.7 in the High Fiber group, while other subtypes were predominantly distributed in the Low Fiber group. **(D)** Cell cycle analysis revealed that Mic.1, Mic.2, Mic.5, and Mic.7 had a higher proportion of cells in S and G2M phases, indicating active proliferation and dynamic transcriptional states. **(E)** Heatmap of representative marker genes highlights the potential molecular identities and functional orientations of each subtype.

Clustering of microglial populations using the Leiden algorithm, followed by marker gene annotation, identified seven transcriptionally and functionally distinct subtypes (Mic.1–Mic.7) (**Figures 8A, B**). Proportional analyses revealed significant enrichment of Mic.5 and Mic.7 in the high-fiber group, while Mic.1, Mic.2, Mic.3, Mic.4, and Mic.6 predominated in the low-fiber group (**Figure 8C**). These distributional shifts suggest that SFDF drives microglia toward reparative and metabolically adaptive phenotypes.

Subtype characterization based on marker gene expression (**Figure 8E**) indicated the following: Mic.1 expressed *Crybb1*, *Angptl7*, and *Cass4*, consistent with a homeostatic maintenance phenotype.Mic.2, expressing *Cmya5*, *Nhsl2*, and *Gm38104*, represented lineage-initiating homeostatic microglia.Mic.3 expressed *Gm12691*, *Macrod2*, and *Gm4759*, suggestive of a transitional state.Mic.4 exhibited high expression of interferon-inducible genes (*Mx1*, *Ifi206*, *Ifit3b*), representing a pro-inflammatory subtype.Mic.5 was enriched for *Pdcd1*, *Cxcl10*, and *Nes*, indicating a stress-activated immunoregulatory phenotype.Mic.6, expressing *Ttr*, *mt-Rnr1*, and *Enpp2*, displayed neurotrophic-like features.Mic.7 showed strong expression of *Fabp5*, *Gpnmb*, and *Atp6v0d2*, marking it as a lipid metabolism–driven reparative subtype closely associated with tissue homeostasis, neuroprotection, and immunosuppression.

Cell cycle analyses further supported functional distinctions (**Figure 8D**). The highest proportions of S-phase cells were observed in Mic.1 (293 cells) and Mic.5 (247 cells), indicating high DNA replication activity. Mic.2 also displayed strong enrichment in G2/M and S phases, consistent with its progenitor identity. Mic.7 exhibited dynamic cycling with substantial G2/M (178) and S-phase (144) fractions and relatively fewer G1-phase cells (113), suggesting a reparative but functionally versatile state. Mic.6, though enriched in the low-fiber group, showed weak cycling activity, consistent with a stable homeostatic role.

Notably, Mic.7 was markedly expanded in the high-fiber group, emerging as the core subtype most functionally reprogrammed by SFDF. This cluster not only highly expressed genes linked to lipid metabolism and immunomodulation but also displayed moderate *Apoe* expression, indicating a potential role as a bridge linking lipid homeostasis with suppression of neuroinflammation.

### 3.9 SFDF intervention reprograms microglial transcriptional profiles toward reparative states

**Figure 9:**
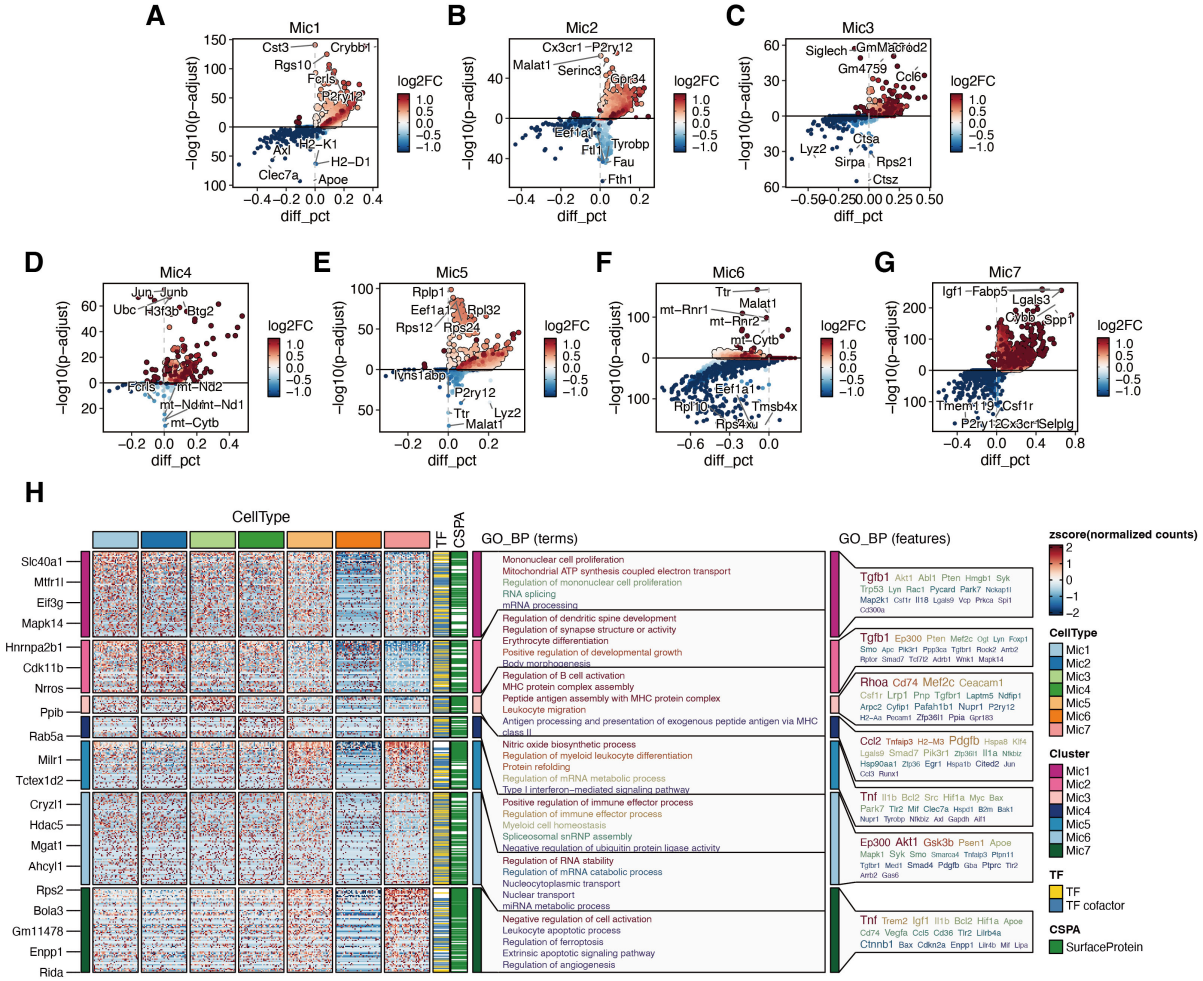
Functional heterogeneity across microglial subtypes revealed by differential expression and GO enrichment analysis. **(A–G)** Volcano plots showing differentially expressed genes in each microglial subtype (Mic.1 to Mic.7). Red and blue indicate significantly upregulated and downregulated genes, respectively (adjusted *P<* 0.05). **(H)** Heatmap summarizing expression of top DEGs across subtypes (left) and enriched GO biological processes (right) for each subtype.

Building on the above findings, we performed differential expression and Gene Ontology (GO) enrichment analyses to further characterize the transcriptional features of each microglial subtype (**Figure 9H**).

Mic.1 expressed *Cst3*, *Crybb1*, *Rgs10*, *Fcrls*, and *P2ry12*, while downregulating *Apoe*, *Clec7a*, *H2-D1*, *Axl*, and *H2-K1*, consistent with a low-inflammatory homeostatic state (**Figure 9A**). GO enrichment indicated roles in monocyte proliferation regulation, mitochondrial energy metabolism, and RNA processing.
Mic.2 expressed *P2ry12*, *Cx3cr1*, *Malat1*, *Serinc3*, and *Gpr34*, confirming a progenitor identity (**Figure 9B**). Downregulated genes included ribosomal and iron metabolism markers (*Fth1*, *Ftl1*), with enrichment in synaptic regulation, developmental growth, and erythroid differentiation, reflecting adaptive support of the neuronal microenvironment.
Mic.3 upregulated *Macrod2*, *Siglech*, *Gm4759*, and *Ccl6*, enriched for antigen processing and MHC class II pathways, including “antigen presentation” and “leukocyte migration” (**Figure 9C**). Downregulated genes such as *Ctsz* and *Sirpa* implicated reduced lysosomal and phagocytic activity, suggesting a transitional immunoregulatory state.
Mic.4 expressed stress-related transcription factors (*Jun*, *Junb*, *Btg2*) and chromatin regulators (*Ubc*, *H3f3b*), while downregulating mitochondrial genes (*mt-Nd2*, *mt-Cytb*) (**Figure 9D**). Enrichment analyses implicated type I interferon signaling, nitric oxide synthesis, and myeloid differentiation, consistent with immune activation.
Mic.5 prominently expressed ribosomal proteins (*Rplp1*, *Rpl32*, *Rps12*, *Rps24*) and translation regulators (*Eef1a1*), with concurrent downregulation of homeostatic markers (*P2ry12*, *Ttr*, *Malat1*) (**Figure 9E**). Pathways included positive regulation of immune effector processes, myeloid homeostasis, and spliceosome assembly, suggesting an active immunoregulatory phenotype.
Mic.6 expressed *Ttr*, *mt-Rnr1*, *mt-Rnr2*, and *Malat1*, enriched in post-transcriptional regulatory pathways, including mRNA stability, miRNA metabolism, and nucleocytoplasmic transport (**Figure 9F**), consistent with a regulatory, neurotrophic-supportive role.
Mic.7, significantly enriched in the SFDF-treated group, was transcriptionally distinct, upregulating key genes related to lipid metabolism, immune suppression, and tissue homeostasis (*Igf1*, *Fabp5*, *Lgals3*, *Cybb*, *Spp1*), while systematically downregulating canonical homeostatic markers (*Tmem119*, *Csf1r*, *P2ry12*, *Cx3cr1*) (**Figure 9G**). GO enrichment highlighted pathways involved in negative regulation of cell activation, leukocyte apoptosis, ferroptosis regulation, and angiogenesis modulation, reflecting a reparative and immune-buffering state.

Together, these results demonstrate that SFDF intervention not only alters microglial subtype composition in the aging brain but also reprograms their transcriptional profiles toward a Mic.7-driven reparative lineage. This remodeling establishes a novel immune homeostatic structure with integrated metabolic regulation, immune modulation, and neuroprotective potential.

### 3.10 SFDF intervention guides the reprogramming of immunoregulatory microglial trajectories

To delineate lineage progression and state transitions among microglial subtypes, we integrated multiple pseudotime trajectory inference methods (Monocle2, Monocle3, Slingshot, and Stavia) to systematically map the dynamic conversion from Mic.2 to Mic.7, while identifying key driver genes and functional pathways.

**Figure 10:**
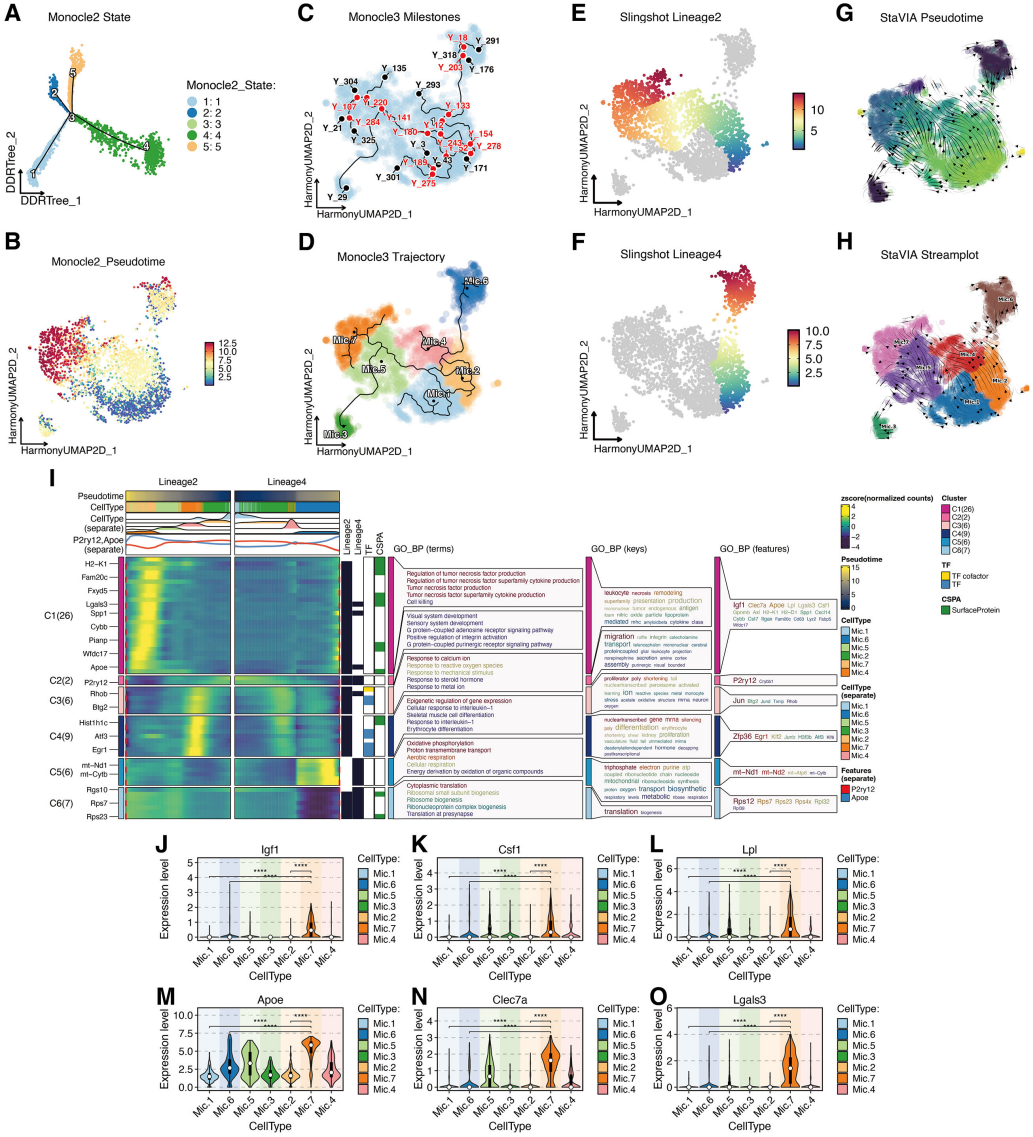
Pseudotime trajectory analysis reveals transitional microglial states. **(A, B)** Monocle2-based trajectory inference visualizing lineage structure and pseudotime progression. **(C, D)** Monocle3 results showing learn_graph-based branching topology in UMAP space. **(E, F)** Slingshot-derived trajectories highlighting Lineage 2 and Lineage 4, indicating distinct differentiation paths. **(G, H)** StAVIA-predicted trajectory structure using state-transition probabilities. **(I)** Heatmap of dynamic gene expression along Slingshot-inferred branches; GO terms indicate key biological functions along Lineage 2. **(J–O)** Violin plots showing expression of representative genes (Igf1, Csf1, Lpl, Apoe, Clec7a, Lgals3) across subtypes with statistical comparisons. *P< 0.05, **P< 0.01, ***P< 0.001, ****P< 0.0001.

In the Monocle2 trajectory (**Figures 10A, B**), Mic.2 was localized at the trajectory root, consistent with a progenitor identity, while Mic.7 aggregated at the terminal pseudotime, suggesting its role as the SFDF-driven terminal state. The topology displayed a branching tree structure, with Mic.4 and Mic.5 occupying transitional nodes and Mic.3 and Mic.6 forming side branches, indicating multipotent differentiation potential.

Monocle3, with enhanced graph learning (**Figures 10C, D**), further defined a primary trajectory extending from Mic.2 to Mic.7, with Mic.3, Mic.4, and Mic.6 contributing secondary branches. This directional continuum underscored Mic.7 as the product of dynamic lineage evolution rather than an isolated phenotype.

Slingshot identified two dominant lineages (Lineage2 and Lineage4) (**Figures 10E, F**). Lineage2 originated from Mic.2, passed through Mic.1, Mic.4, and Mic.5, and terminated in Mic.7, clearly outlining the Mic.2-to-Mic.7 transition. Lineage4 primarily branched toward Mic.6, with little contribution from Mic.7, highlighting its distinct terminal trajectory.

Stavia further integrated state-transition probabilities to generate high-resolution pseudotime flow maps (**Figures 10G, H**). Mic.2 occupied the central convergence hub, while Mic.7 consistently emerged at the terminal output, reinforcing its status as the SFDF-induced end state. Consensus across all four algorithms strongly supported a primary Mic.2-to-Mic.7 differentiation trajectory.

Gene expression dynamics along Lineage2 revealed a cluster of upregulated genes (Cluster 1; **Figure 10I**) encompassing:

1. Immunoregulation: *Clec7a*, *Apoe*, *Csf1*, *Cxcl14*, *Cd63*
2. Tissue repair and remodeling: *Igf1*, *Spp1*, *Lgals3*, *Lpl*, *Gpnmb*
3. Phagocytosis and oxidative stress response: *Cybb*, *Cst7*, *Lyz2*, *Fabp5*
4. Antigen presentation: *H2-K1*, *H2-D1*
5. Cell adhesion and migration: *Axl*, *Itgax*, *Wfdc17*, *Fam20c*

GO enrichment (**Figure 10I**) highlighted regulation of TNF-family cytokine production, immune response modulation, and apoptotic pathways. These findings indicate that the Mic.2-to-Mic.7 transition is not a simple activation sequence but involves the coordinated establishment of an immunoregulatory, phagocytic, and metabolically adaptive program.

Violin plots of six key Cluster 1 genes (*Igf1*, *Csf1*, *Lpl*, *Apoe*, *Clec7a*, *Lgals3*) confirmed significantly higher expression in Mic.7 compared with Mic.2 and Mic.6 (P< 0.01; **Figures 10J–O**). Notably, *Igf1* and *Csf1* emerged as Mic.7-defining markers, supporting its role as a reparative and regulatory terminal state.

Collectively, multi-algorithm trajectory analyses consistently demonstrated that under SFDF intervention, microglia evolve from progenitor Mic.2 to terminal Mic.7, accompanied by upregulation of functional gene clusters involved in immune modulation, apoptotic regulation, and metabolic adaptation. Mic.7 thus represents a reparative-regulatory subtype capable of reprogramming the aging brain microenvironment and counteracting age-associated immune imbalance.

### 3.11 Co-expression modules and metabolic reprogramming of microglia under SFDF intervention

**Figure 11:**
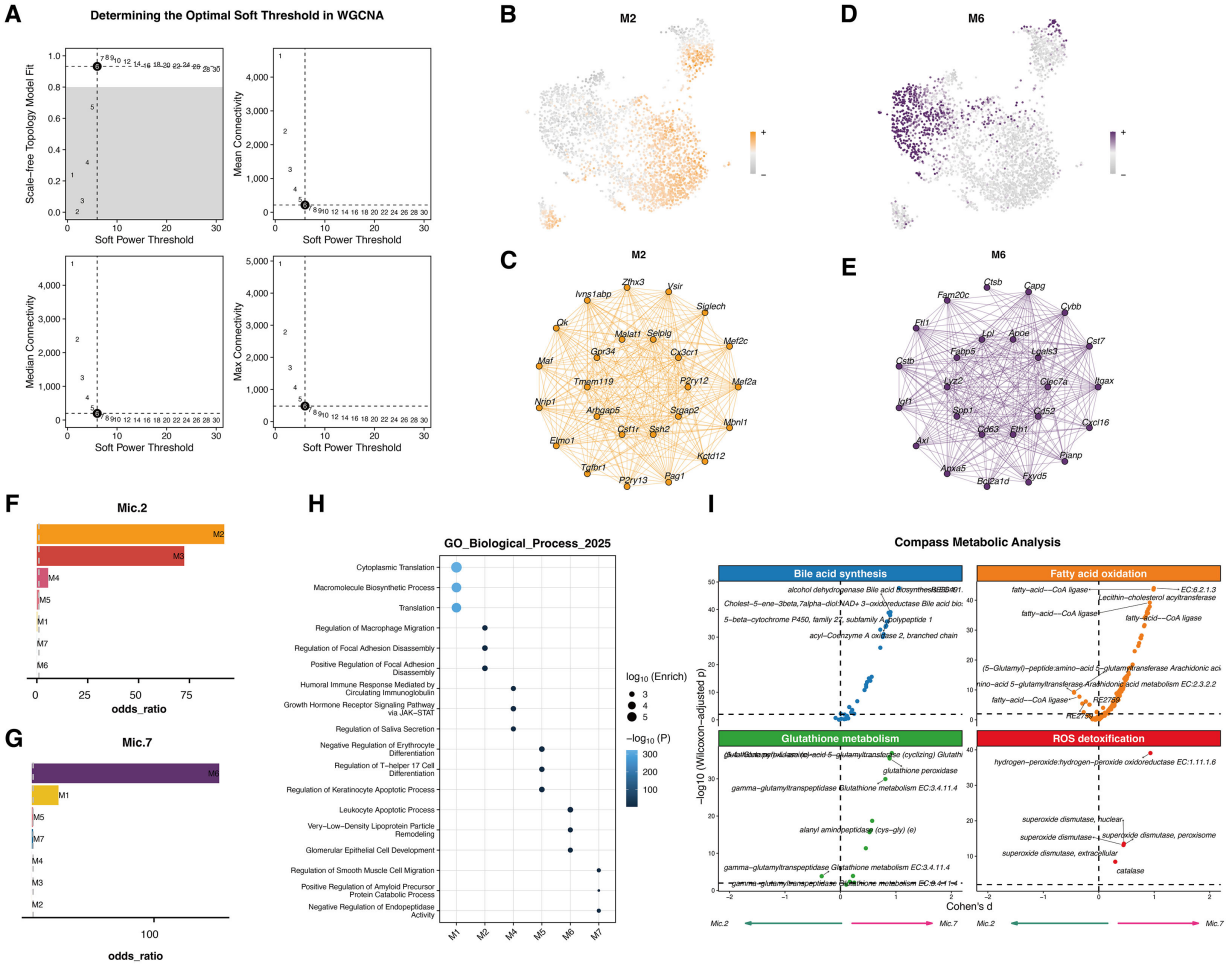
Co-expression modules and metabolic reprogramming in microglial subtypes. **(A)** Scale-free topology model fit curve for determining soft-thresholding power in WGCNA. **(B, C)** AUCell activity projection (UMAP) and top 20 hub gene network of module M2, predominantly enriched in homeostatic microglia. **(D, E)** AUCell activity map and top hub gene network of module M6, mainly associated with Mic.7 cells. **(F, G)** Odds ratio analysis of module enrichment within Mic.2 **(F)** and Mic.7 **(G)** subpopulations. **(H)** GO enrichment analysis of all modules, highlighting functional differences such as immune regulation, apoptosis, and lipid metabolism. **(I)** Compass-inferred metabolic activity differences between Mic.2 and Mic.7, showing enhanced pathways in Mic.7 including bile acid metabolism, fatty acid activation, glutathione metabolism, and ROS detoxification.

To further elucidate how SFDF drives the transition from Mic.2 to the functionally active Mic.7 state, we applied high-dimensional weighted gene co-expression network analysis (hdWGCNA) (**Figure 11A**) and integrated metabolic modeling via Compass.

We identified multiple co-expression modules, of which M2 and M6 displayed marked subtype specificity (**Figures 11B–E**). The M2 module, enriched in Mic.2, included *P2ry12*, *Cx3cr1*, and *Csf1r*, representing homeostatic programs essential for surveillance, neuronal contact, and environmental integration. GO enrichment linked M2 to adhesion, positional maintenance, and signal regulation, confirming Mic.2 as a quiescent “sentinel” state.

In contrast, the M6 module was strongly enriched in Mic.7, with core genes *Igf1*, *Apoe*, *Fabp5*, *Lgals3*, and *Spp1*. GO analysis (**Figure 11H**) revealed enrichment in leukocyte apoptosis regulation, VLDL remodeling, and neurodevelopmental processes, highlighting Mic.7’s immunoregulatory and metabolic functions. Module enrichment analysis confirmed that Mic.2 predominantly expressed M2 while Mic.7 selectively expressed M6 (**Figures 11F, G**), underscoring a wholesale transcriptional program shift rather than isolated gene changes.

Compass metabolic modeling (**Figure 11I**) demonstrated marked enhancement of Mic.7 metabolic capacity, particularly in bile acid metabolism, fatty acid β-oxidation, and glutathione-ROS homeostasis.

1. Bile acid metabolism was significantly upregulated, suggesting anti-inflammatory signaling potential via FXR/TGR5 pathways.
2. Fatty acid activation and β-oxidation were elevated, supporting enhanced lipid uptake, processing, and energy generation, consistent with high expression of *Apoe*, *Lpl*, and *Fabp5*.
3. Glutathione cycling and ROS clearance were strongly activated, with genes such as *Cybb*, *Cst7*, and *Gpnmb* upregulated, reflecting a robust antioxidative defense system.

Together, the transition from Mic.2 to Mic.7 represents a coordinated systems-level reprogramming encompassing module replacement, metabolic adaptation, and functional redefinition. This reprogramming underlies SFDF’s capacity to restore microglial immune homeostasis in the aging brain.

### 3.12 Transcription factor regulatory networks define Mic.7-specific programs

**Figure 12:**
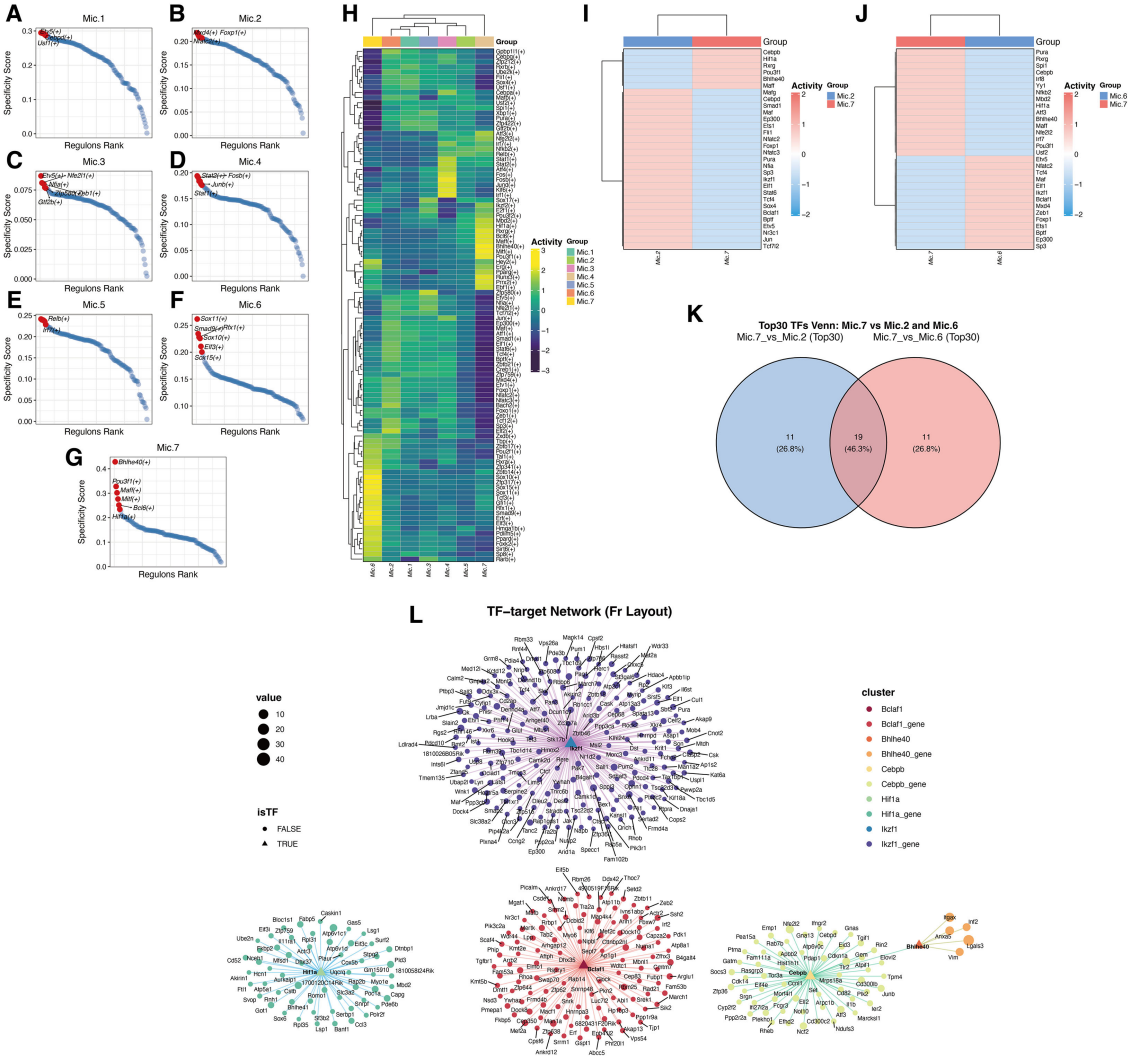
Single-cell transcription factor regulatory network analysis reveals microglial subtype-specific regulatory features. **(A–G)** Top-ranked transcription factor regulons inferred by pySCENIC in each microglial subtype (Mic.1–Mic.7), based on co-expression and motif enrichment analysis. **(H)** Heatmap of regulon activity calculated using the AUCell algorithm, showing global heterogeneity in transcriptional regulation across subtypes. **(I, J)** Differential regulon activity heatmaps between Mic.7 and Mic.2 **(I)**, and between Mic.7 and Mic.6 **(J, K)** Venn diagram showing the overlap of the top 30 transcription factors among Mic.7, Mic.2, and Mic.6. **(L)** Regulatory network constructed from five core transcription factors and their predicted target genes.

To uncover the regulatory mechanisms supporting Mic.7 identity, we performed transcription factor (TF) regulatory network inference using pySCENIC across all seven microglial subtypes (**Figures 12A–G**).

1. Mic.1 and Mic.2 were enriched for TFs including *Etv5*, *Cebpd*, *Mxd4*, *Foxp1*, and *Nfatc2*, consistent with homeostatic maintenance and anti-inflammatory balance.
2. Mic.3 exhibited activity of *Nfe2l1* and *Zeb1*, linked to immune transition states.
3. Mic.4 and Mic.5 were dominated by inflammatory TFs (*Stat1/2*, *Junb*, *Relb*, *Irf7*), consistent with immune activation.
4. Mic.6 showed activity of Sox family TFs (*Sox11*, *Sox10*, *Sox15*), *Elf3*, and *Smad9*, reflecting integration of neurodevelopmental signaling.

Strikingly, Mic.7 formed a distinct TF network, enriched for *Bhlhe40*, *Maff*, *Bcl6*, *Mitf*, *Pou3f1*, and *Hif1a* (**Figure 12G**). These TFs exhibited subtype-specific activation, defining Mic.7’s unique transcriptional identity (**Figure 12H**). Heatmaps highlighted clear divergence between Mic.7 and both Mic.2 (**Figure 12I**) and Mic.6 (**Figure 12J**).

Venn analysis (**Figure 12K**) identified five shared TFs—*Bhlhe40*, *Maff*, *Bcl6*, *Mitf*, and *Hif1a*—as core regulators distinguishing Mic.7 from both Mic.2 and Mic.6. Their target network (**Figure 12L**) included *Lgals3*, *Spp1*, *Gpnmb*, and *Fabp5*, all previously identified as Mic.7 signature genes in co-expression, GO, and Compass analyses. Although *Apoe* was not a direct target in this network, its high expression in Mic.7 suggests regulation via indirect or secondary TF interactions.

Thus, SFDF intervention induces a Mic.7-specific TF regulatory module centered on *Bhlhe40*, *Maff*, *Bcl6*, *Mitf*, and *Hif1a*. This network orchestrates immune suppression, metabolic adaptation, and stress response, providing transcriptional support for the reparative-regulatory Mic.7 phenotype and highlighting a core mechanism by which SFDF restores immune homeostasis in the aging brain.

## 4 Discussion

This study integrates nationally representative data from the NHANES cohort, experimental evidence from a D-galactose–induced accelerated aging mouse model, and single-cell transcriptomic analyses of brain tissue from naturally aged mice to systematically evaluate the relationship between total dietary fiber intake and cognitive function in older adults. We further explored the underlying biological mechanisms from behavioral phenotypes to microglial lineage remodeling. Importantly, because the NHANES component is cross-sectional, findings from this part of the study should be interpreted as associations rather than causal effects; causal language is reserved for the experimental components, and mechanistic interpretations are presented as hypotheses supported by the animal and single-cell data.

In cross-sectional analyses, we observed a consistent positive association between total dietary fiber intake and multiple dimensions of cognitive function. The strongest associations were found for the CERAD Instant Recall and Animal Fluency tests, suggesting that higher fiber intake is linked to better performance across several cognitive domains. These findings are broadly consistent with prior NHANES-based analyses linking higher dietary fiber intake to better cognitive performance in older adults, although the specific cognitive tests showing the strongest associations and the shape/inflection of dose–response curves have differed across studies, likely due to differences in exposure definitions, analytic approaches, and covariate adjustment (18, 57). Notably, CERAD Instant Recall, a sensitive measure of short-term memory and hippocampal function, was most responsive to total dietary fiber intake. While the NHANES results alone cannot determine mechanism, this pattern is compatible with the hypothesis that fiber-related differences in systemic inflammation, oxidative stress, and/or metabolic status may translate into more favorable conditions for hippocampal-dependent cognition—a hypothesis that is further supported by our experimental findings. The association with Animal Fluency, which reflects semantic retrieval and prefrontal executive function, indicates that higher fiber intake is also associated with better performance in higher-order cognitive processes (58). Improvements observed in the DSST, which captures attention, processing speed, and working memory, may similarly reflect fiber-associated differences in integrative neural networks and cardiometabolic or inflammatory profiles (59). In contrast, CERAD Delayed Recall exhibited weaker associations, possibly due to its sensitivity to long-term memory retention and age-related decline, and/or greater measurement variability and ceiling/floor effects compared with immediate recall. Together, these domain-specific patterns suggest that fiber intake may be more strongly associated with cognitive processes that are particularly vulnerable to aging-related inflammatory-metabolic stress, although longitudinal data are required to confirm temporal ordering and test domain-specific trajectories.

Dose–response analyses revealed a characteristic nonlinear “rapid-gain to plateau” pattern in CERAD Instant Recall and Animal Fluency scores, with approximately 15 g/day emerging as a threshold for cognitive benefit. This consistent pattern across cognitive domains underscores its public health relevance, particularly because many older adults remain well below recommended fiber intake levels. Although dietary guidelines in both the U.S. and China recommend 25–30 g/day of fiber, actual intake remains substantially below this target (60, 61). Our findings suggest that even modest increases in total dietary fiber intake—especially among individuals with low baseline intake—are associated with appreciable differences in cognitive performance. This “low-dose, high-response” feature holds practical value for designing feasible dietary strategies, especially among older adults, and may help prioritize interventions that focus on shifting individuals out of very low intake ranges. Subgroup analyses showed that while sex, education, and smoking status influenced the shape of the response curves, none significantly modified the main effect, reinforcing the robustness and generalizability of the findings. Nonetheless, because these are observational dose–response relationships, the plateau pattern may reflect residual confounding, clustering of healthier behaviors at higher intake levels, or measurement error in dietary assessment, and thus should be interpreted cautiously. Notably, prior NHANES spline analyses have also suggested plateauing at higher intake ranges for certain tests (e.g., DSST in some reports), underscoring that the apparent “threshold” may be test-specific and context-dependent (18).

These results align with multiple high-quality prospective studies. For instance, a Japanese cohort reported an inverse association between total dietary fiber intake and dementia risk (62). Consistent associations between higher total dietary fiber intake and reduced cognitive decline have been reported across diverse populations, with particularly strong evidence in Asian and North American cohorts (63–65). A recent UK Biobank study further supports the broader concept that dietary inflammatory potential is related to subsequent brain disorders, which is relevant because higher-fiber dietary patterns are often associated with lower inflammatory profiles (66). Our findings are directionally consistent with this literature and extend it by characterizing a nonlinear exposure–outcome pattern using advanced modeling, providing additional granularity on how cognitive performance varies across the intake distribution. At the same time, differences across cohorts in dietary assessment methods, cognitive endpoints, and covariate adjustment strategies can contribute to heterogeneity, underscoring the need for well-designed longitudinal and interventional studies to validate the dose range and confirm whether threshold-like effects translate into causal benefit.

Building on population findings, we employed a D-galactose-induced accelerated aging mouse model to test the causal role of SFDF. SFDF intervention significantly improved learning and memory performance, alleviated anxiety-like behavior, and enhanced locomotor activity. These effects were accompanied by reduced expression of pro-inflammatory and pro-oxidative markers (IL-6, TNF-α, MDA) and increased levels of anti-inflammatory and antioxidant factors (IL-10, SOD, GSH), suggesting a neuroprotective mechanism in this model that is mediated, at least in part, through suppression of neuroinflammation and mitigation of oxidative stress. The experimental results therefore provide causal evidence (in mice) that SFDF supplementation can improve neurobehavioral outcomes under controlled conditions, strengthening biological plausibility for the NHANES associations while not, by itself, proving causality of habitual intake in humans. Previous studies have established that SCFAs—key metabolites of fiber fermentation—can cross the blood–brain barrier, regulate neuroimmune signaling (67, 68), and promote synaptic plasticity and neurogenesis (69, 70). In addition, experimental work in aging mice has shown that increasing SFDF (e.g., inulin) and/or butyrate can attenuate microglial pro-inflammatory profiles and improve neuroinflammation-related phenotypes, providing mechanistic plausibility for soluble-fiber effects on brain aging (37). Emerging randomized evidence in older adults further suggests that prebiotic-driven gut microbiome modulation may improve cognition over a 12-week intervention window, supporting translational relevance for the gut–brain axis hypothesis (71). Our animal findings align with these pathways and support a role for the gut–brain axis in mediating fiber-induced cognitive benefits. However, because we did not directly measure gut microbiota composition, SCFA levels, or barrier integrity in this work, these mechanistic links should be viewed as testable hypotheses supported by convergent evidence rather than definitive mediators in the current dataset.

At the molecular level, single-cell RNA sequencing identified Mic.7, a microglial subtype highly responsive to SFDF. Mic.7 was enriched for genes such as Igf1, Apoe, Fabp5, and Lgals3, associated with tissue repair, phagocytosis, and metabolic homeostasis, reflecting a reparative and immunoregulatory phenotype (72–74). Pseudotime trajectory analysis revealed that Mic.7 arises from the homeostatic Mic.2 lineage, acquiring specialized functions during its transition. Its transcriptional profile overlapped with reparative microglia previously reported in Alzheimer’s disease and white matter lesion models, suggesting that Mic.7 may represent an adaptive microglial program that supports tissue maintenance in response to stressors. This interpretation is consistent with foundational evidence that gut microbiota and microbiota-derived SCFAs regulate microglial maturation and homeostatic function, offering a plausible upstream link between fermentable substrates and microglial state remodeling (75). Compass metabolic modeling further demonstrated that Mic.7 exhibits enhanced activity in fatty acid activation, bile acid metabolism, glutathione synthesis, and ROS clearance, suggesting a central role in oxidative stress defense and neuroprotection. More broadly, growing literature highlights microglial metabolic reprogramming and redox regulation as key determinants of inflammatory phenotypes and disease-relevant microglial states in neurodegeneration (76). Strikingly, Mic.7 was significantly enriched in the SFDF group but depleted in naturally aged mice, highlighting it as a nutrient-responsive lineage with strong environmental sensitivity. Because the single-cell analyses are observational with respect to microglial states and diet, we interpret Mic.7 primarily as a candidate cellular correlate and mechanistic node rather than a confirmed mediator; future functional experiments are needed to determine whether inducing Mic.7-like programs is necessary or sufficient for the behavioral benefits observed.

Our study advances prior mechanistic research by identifying the central cellular target and pathway of SFDF intervention. Earlier work has shown that fructooligosaccharides alleviate Alzheimer’s-related cognitive decline via the microbiota–gut–brain axis (77), and inulin supplementation modulates gut microbiota to mitigate age-related cognitive deficits (78). However, neither study identified the central mediating cell type. Here, we demonstrate at single-cell resolution that Mic.7 serves as the key SFDF-responsive microglial lineage, providing a more specific cellular hypothesis linking diet to neuroimmune remodeling. Notably, Mic.7-expressed molecules such as Igf1 and Lgals3 may be quantifiable in peripheral blood or cerebrospinal fluid, offering translational potential as biomarkers of nutritional responsiveness. This opens the possibility that future trials could incorporate biomarker-informed stratification to identify subgroups most likely to respond to fiber-based interventions and to monitor biological engagement of the proposed pathway.

Methodologically, this study has several strengths. First, we employed multidimensional cognitive batteries, avoiding the limitations of single metrics such as MMSE. Second, the use of restricted cubic spline models enabled systematic evaluation of marginal effects across continuous exposure ranges, identifying optimal intervention thresholds (79). Third, our models rigorously adjusted for sociodemographic, behavioral, and metabolic covariates, enhancing robustness and validity. Integration with animal experiments and single-cell analyses allowed us to build a multilayered evidence framework, spanning population observation to mechanistic cellular insights. This triangulation—association in humans, causal supplementation effects in animals, and cell-type-specific correlates in aging brain—strengthens confidence that the overall signal is biologically meaningful and worthy of further testing in longitudinal and interventional settings.

Nevertheless, several limitations should be noted. The NHANES data are cross-sectional, limiting causal inference and leaving open the possibility of reverse causation (e.g., poorer cognition influencing diet quality), although animal experiments strengthen causal plausibility for supplementation effects. Dietary assessment relied on 24-hour recalls, which may be affected by recall bias and day-to-day variation; future studies should incorporate multi-day recalls, food frequency questionnaires, and objective biomarkers such as SCFA levels to improve accuracy. In addition, residual confounding cannot be fully excluded, including unmeasured components of dietary pattern, food preparation capability, comorbidity severity, medication use, and broader socioeconomic determinants. For mechanistic resolution, the present work did not directly quantify microbiome composition, SCFAs, gut permeability, or blood–brain barrier integrity, which limits our ability to pinpoint upstream mediators connecting fiber to neuroinflammatory phenotypes. Additionally, the mouse experiments used purified inulin/SFDF, whereas human fiber sources are more diverse (e.g., grains, legumes, vegetables), which may yield different fermentation and metabolic profiles. Finally, while Mic.7 is strongly implicated as a responsive microglial subtype, confirmation of causality will require targeted perturbation of microglial programs and longitudinal mapping of microglial state transitions under dietary manipulation.

In conclusion, this study provides convergent evidence across population, animal, and single-cell levels that dietary fiber—particularly soluble fractions—is closely linked to cognitive function in older adults and has neurobehavioral benefits under experimental conditions. Population analyses established robust positive associations across multiple cognitive domains; animal models confirmed SFDF’s causal benefits on neurobehavioral outcomes, neuroinflammation, and oxidative stress; and single-cell analyses identified the reparative microglial subtype Mic.7 as a strong candidate cellular node related to these effects. Together, these findings deepen mechanistic understanding and provide a translational framework for developing personalized nutritional strategies aimed at delaying cognitive decline and neurodegeneration in aging populations, while highlighting the need for longitudinal studies and randomized trials to confirm causality and define optimal fiber types and dose ranges.

## Data Availability

The NHANES datasets analyzed in this study are publicly available from the National Center for Health Statistics (NCHS) at https://www.cdc.gov/nchs/nhanes/. The snRNA-seq data used in this study were obtained from the Gene Expression Omnibus (GEO) under accession number GSE163055. The R code required to reproduce the main statistical results will be made publicly available on GitHub at https://github.com/aotai12138/NHANES-Fiber-Cognition-Analysis-Code.git. Other data generated or analyzed during this study are available from the corresponding author upon reasonable request

## Glossary

AMPM: Automated Multiple-Pass Method
ARCI: Aging-related cognitive impairment
AUCell: Area Under the Curve Cell scoring
Apoe: Apolipoprotein E
Bhlhe40: Basic helix-loop-helix family member e40
BMI: Body mass index
CERAD: Consortium to Establish a Registry for Alzheimer’s Disease
Csf1: Colony stimulating factor 1
Csf1r: Colony stimulating factor 1 receptor
Cx3cr1: CX3C chemokine receptor 1
Cybb: Cytochrome b-245 beta chain
DNA: Deoxyribonucleic acid
DSST: Digit Symbol Substitution Test
ELISA: Enzyme-linked immunosorbent assay
Fabp5: Fatty acid binding protein 5
GEO: Gene Expression Omnibus
GO: Gene Ontology
GRN: Gene regulatory network
GSH: Glutathione (reduced form)
GSSG: Glutathione disulfide (oxidized form)
Hif1a: Hypoxia-inducible factor 1-alpha
HVGs: Highly variable genes
Igf1: Insulin-like growth factor 1
IL-1β: Interleukin-1 beta
IL-6: Interleukin-6
IL-10: Interleukin-10
Lgals3: Galectin-3
Lpl: Lipoprotein lipase
Maff: MAF bZIP transcription factor F
MDA: Malondialdehyde
Mitf: Microphthalmia-associated transcription factor
MWM: Morris Water Maze
NCHS: National Center for Health Statistics
NHANES: National Health and Nutrition Examination Survey
OFT: Open Field Test
PIR: Poverty Income Ratio
RCS: Restricted cubic spline
ROS: Reactive oxygen species
SCENIC: Single-Cell Regulatory Network Inference and Clustering
SCFAs: Short-chain fatty acids
SFDF: Soluble fermentable dietary fiber
snRNA-seq: Single-nucleus RNA sequencing
SOD: Superoxide dismutase
Spp1: Secreted phosphoprotein 1
TF: Transcription factor
TNF-α: Tumor necrosis factor-alpha
TOM: Topological overlap matrix
Ttr: Transthyretin
UMAP: Uniform Manifold Approximation and Projection
WT: Wild type
